# Bornlisy Attenuates Colitis-Associated Colorectal Cancer *via* Inhibiting GPR43-Mediated Glycolysis

**DOI:** 10.3389/fnut.2021.706382

**Published:** 2021-11-12

**Authors:** Xia Lu, Shuping Qiao, Chen Peng, Wenyue Yan, Zhen Xu, Junxing Qu, Yayi Hou, Shuli Zhao, Ping Chen, Tingting Wang

**Affiliations:** ^1^The State Key Laboratory of Pharmaceutical Biotechnology, Division of Immunology, Medical School, Nanjing University, Nanjing, China; ^2^Department of Oncology, Yancheng First Hospital, Affiliated Hospital of Nanjing University Medical School, The First People's Hospital of Yancheng, Yancheng, China; ^3^General Clinical Research Center, Nanjing First Hospital, Nanjing Medical University, Nanjing, China

**Keywords:** colitis-associated colon cancer, probiotic, metabolism reprogramming, glycolysis, GPR43

## Abstract

There is evidence that probiotics have a broad antitumor effect in colorectal cancer (CRC). However, the mechanism remains obscure. Here, we investigated the effect of Bornlisy (BO)-cocktails of three probiotics on colitis-associated colon cancer (CAC) and the underlying mechanism. The treatment of CAC mice with BO resulted in decreased tumor loads as compared with their counterparts. BO also inhibited the proliferation and metastasis of CRC cells *in vitro*. Furthermore, BO inhibited cell proliferation through downregulating glycolysis. Activating glycolysis reversed the protective role of BO in the CAC mice. Mechanically, BO administration promoted the activation of GPR43, followed by its downstream PLC-PKC-ERK pathway, which led to decreased glucose metabolism. These results suggest that BO may provide an intervention strategy for CRC therapy, while GPR43 is a potential targeting receptor during the BO treatment.

## Introduction

Globally, colorectal cancer (CRC) is the fourth most common non-cutaneous malignancy and the second most frequent cause of cancer-related death (1). Although there are dramatic improvements in the CRC treatment with surgical technique in the past decades, the enhancement of the 5-year relative survival rate for the patients with CRC is not significant (2, 3). Accordingly, it is urgent for us to find a new way to deal with this disease.

As found in more and more research recently, the gut microbiota plays a critical role for its host in health maintenance and disease pathogenesis (4–6). Consequently, the strategy through the regulation of gut microbiota has been thought to be a promising therapy to treat digestive diseases, particularly colon cancer (7–9). It was demonstrated that fecal microbiota transplant (FMT), which transfers the feces from a healthy human donor to an affected subject, can be effectively used in the clinical therapy of cancer (10, 11). However, the development of FMT has been impeded by the operation without standardized protocol, and pathogenic bacteria persisting inevitably, which brings unexpected damage to the patients (12). The most frequently used food supplements among the different kinds of bacterial strains are probiotics, which are considered safe and produced with the standardized protocol completely (13). A wide variety of disorders have been shown to respond positively to the probiotics, such as type 2 diabetes (14), Alzheimer's disease (15), allergic rhinitis (16), metabolic syndrome (17), intestinal inflammation (18, 19), and cancer (20–22). Nowadays, probiotics have been found to exerts a tumor-suppressive effect (23), such as colon cancer (24). For example, in a prospective intervention study, after administered *Lactobacillus acidophilus NCFM* and *Bifidobacterium lactis Bl-04*, the microbial profile in the patients with CRC altered (25). As a probiotic with the capability of producing butyrate, *Clostridium butyricum* can regulate the gut microbiota and Wnt signaling to repress the development of intestinal tumors, which show the promising role of the butyrate-producing bacteria to fight against CRC (26). In colon cancer cells, the induction of DNA damage-inducible transcript 3 (DDIT3), which is associated with C-Jun N-terminal Kinase (JNK), mediates the process for ferrichrome to induce cell apoptosis, and notably, the ferrichrome is produced by *Lactobacillus casei ATCC334* (27). The extensive antitumor performance of probiotics has been found in number of studies, but we still know little about the particular mechanism.

Metabolic reprogramming is widely observed during cancer development to confer the cancer cells and the ability to survive and proliferate (28, 29). Almost all the energy obtained by the normal cells is produced from the reaction of oxidative phosphorylation in mitochondrial, however, the cancer cells obtain their primary energy from aerobic glycolysis, which is quite different from the normal cells. This course is named the “Warburg effect” (30–32). As one of the most actively deregulated oncogenes, MYC is expected to mediate the expression of 15% of total genes (33), including various metabolic genes (34). MYC is able to upregulate the genes which express the glucose transporter 1(GLUT1) at the transcriptional level (35), lactate dehydrogenase A (LDHA) (36), hexokinase 2(HK2) (37), and pyruvate kinase isoform 2 (PKM2) (38), consequently, in pace with the glucose intake increasing as well as the glucose converting to lactate fast, MYC enhances the reaction of glycolysis. Although the fact that the activity of glycolysis is increased in the CRC has been verified, the role that probiotics performed in the change of glucose during its metabolic process in the CRC, is still seldom studied.

Dietary fiber without being digested is fermented by the colonic microbial to produce primarily short-chain fatty acids (SCFAs), whose main components are acetate, propionate, and butyrate (39, 40). As the seven transmembrane receptors, G protein-coupled receptors (GPCRs) take part in the activation of heterotrimeric G protein. Because these GPCRs take part in many diseases, the receptors provide lots of recognition sites for the therapeutic utilization for many different kinds of diseases (41). Up to now, due to the important physiological performance in different kinds of biological reactions, four free fatty acid receptors (FFARs), FFAR1 (GPR40), FFAR2 (GPR43), FFAR3 (GPR41), and FFAR4 (GPR120) have attracted significant attention (42, 43). The SCFAs have potential anti-inflammatory and anti-carcinogenic properties (44). The SCFAs repress the activity of histone deacetylase (HDAC) at the Foxp3 locus to promote the regulatory T cells (Treg) in the colon (39, 45). In patients with colon cancer, the expression of GPR109A and GPR43, which belong to the SCFAs receptors, decreased significantly (46, 47). In the patients with inflammatory bowel disease (IBD) or colon cancer, the amount of the bacteria with the capacity of producing butyrate in the gut mucosa and in the feces samples was found to decrease (48, 49). However, there are few reports on whether the GPRs signal activated by the SCFAs affects the metabolism of CRC.

Here, we evaluated the effect of a novel probiotic mixture Bornlisy (BO) on the development of CAC. Our results show that the BO treatment inhibits tumorigenesis and glucose metabolism *via* activating GPR43 in the CRC. From a broad perspective, our recent research shows that BO exhibits remarkable and promising therapeutic performance in the clinical treatment of CRC.

## Materials and Methods

### Bacteria Culture and Preparation of BO

*Lactobacillus acidophilus* (ATCC 33198), *L*. (ATCC 11842), *B. subtilis* (ATCC 6051) were purchased from China General Microbiological Culture Collection Center (CGMCC), Beijing, China. These bacteria were cultured in the specific liquid medium composed of 1% honey, 4% brown sugar, and water at 37°C. *E. coli* (ATCC 25922) was purchased from CGMCC and was cultured in the Luria-Bertani (LB) medium at 37°C. BO is fermented by mixing *L. acidophilus, L. bulgaricus*, and *B. subtilis* on a 1:1:1 scale in the specific liquid medium at 37°C. After 24 h, the fermentation of a single strain and the probiotic mixture were collected. Before intragastric administration (i.g.), the fermentation mixture of the three probiotics was dissolved in ddH_2_O at a concentration of 10^8^ CFU/ml.

### Animals and Mouse Model

The female C57BL/6J mice aged 6–8 weeks were fed in a cage with five mice in each cage. The mice were fed for 12/12 h in a day-night cycle under the control of humidity (50 ± 5%) and temperature (22 ± 2°C). The animal study was reviewed and approved by the National Institutes of Health guide for the care and use of laboratory animals, as well as the Institutional Animal Care and Use Committee at Nanjing University, China.

For a generation of the CAC model, the mice were injected intraperitoneally with AOM (10 mg/kg; # A5486; Sigma-Aldrich, MO, USA) on the first day. One cycle consists of 7 days of DSS followed by 14 days of water. The mice were orally inoculated with BO (10 mL/kg) once every 2 days during the first, fourth, and seventh weeks. For the glycolysis activation experiments, FBP (Sangon Biotech, Shanghai, China, #81028-91-3, 500 mg/kg, i.p. once every 2 days) was added for three cycles in the CAC model. The mice were euthanized on day 100. On the 100th day, the colons were removed and collected.

### Cell Lines and Cell Culture

The CRC cell lines (CT26 and CT116) were obtained from the American Type Culture Collection (ATCC; Shanghai, China). A cell was cultured in DMEM (Gibco, USA) supplemented with 10% FBS (Gibco, USA), 1% penicillin, and 1% streptomycin (Gibco, USA) at 37°C in a 5% CO_2_-humified atmosphere.

### Acid and Bile Acid Tolerance Test

The single strain and fermentation mixture were inoculated in the specific liquid medium with 5% inoculum at pH 1.0, 2.0, 3.0, 4.0, 5.0, and 6.0, respectively, and were cultured at 37°C for 3 h at 100 r/min, and optical density (OD) 600 was measured by spectrophotometer.

The single strain and fermentation mixture were inoculated in the specific liquid medium containing 0.1, 0.2, 0.3, 0.4, 0.5, and 0.6% of bovine bile salt at 5% inoculation amount, respectively, and were cultured at 37°C for 3 h at 100 r/min, and their OD 600 was measured by spectrophotometer.

### Antibacterial Assay

*Escherichia coli* was inoculated on the LB solid medium for the night at 37°C for 24 h. Some 9 mm holes were prepared. Antibacterial assay of the single strain and fermentation mixture was carried out by the Agar disk diffusion method. The zones of inhibition were measured.

### Histopathological and IHC Analyses

The colonic tissue specimens were embedded in paraffin, sectioned, and stained with hematoxylin and eosin. The tumor severity was assessed by a pathologist who did not know the experimental design using the table of pathological scores (50) (Supplementary Table 1). For IHC staining, the tumor samples were stained with the antibodies of Ki-67 (Cell Signaling, USA, #12202) and PCNA (Cell Signaling, USA, #13110). The stained sections were examined under a light microscope.

### Cell Proliferation Assay

The cell proliferation was detected using a CCK8 (Dojindo Laboratories, Japan). The cell suspensions (3 × 10^3^/well) were seeded in the 96-well-culture plates. The CCK8 solution (10 μl) was added to each well, and the cells were cultured for 2 h at 37°C. Then, the absorption was evaluated by a microplate reader at 450 nm (Tecan, Switzerland).

### EdU Assay Cells

The cell suspensions (1 × 10^4^/well) were seeded on the 96-well culture plates and cultured for 24 h. A Cell-Light EdU Apollo567 *In Vitro* Kit (Ribobio, China) was used to detect the incorporated EdU according to the protocols from the manufacturer. The staining results were observed under the fluorescence microscope.

### Wound Healing Scratch Assay

The cell suspensions (6 × 10^5^/well) were seeded into the 6-well-plates. When cell confluence reached ~90–100%, three vertical scratches were engraved on the cells using a 200 μl tip. Then, the cells were washed with PBS solution three times and cultured in the 37°C-cell incubator. After 0 and 24 h, the breadths of scratches were measured. The percentage of migration was calculated as follows: [(the breadths of scratches at 0 h –the breadths of scratches at 24 h)/the breadths of scratches at 0 h] ×100%.

### Transwell Migration Assay

A 24-well cell culture inserts containing a PET membrane (8.0-μm pore size, #353097; BD Biosciences, NJ, USA) was used in the transwell migration assay. The upper compartment was supplemented with 200 L serum-free DMEM cell suspension (2 × 10^5^ cells), and the lower compartment was supplemented with 800 L DMEM (10% FBS). After incubation for 24 h, the migrated cells at the bottom of the membrane were fixed with 4% PFA for 20 min and stained with 0.1% crystal violet for further analysis.

### RNA Isolation and Quantitative Real-Time PCR (qPCR)

RNA extraction from the cells and tissues was isolated using TRIzol. cDNA was obtained by a Takara PrimeScript RT reagent kit (Takara Bio Inc., Japan). Quantitative real-time PCR (qPCR) was performed using the SYBR Green PCR Master Mix (Invitrogen, MA, USA) The sequences of primers used in the study are shown in Supplementary Table 2.

### Protein Extraction and Western Blotting

A protein from the tissues and cells was extracted using the bicinchoninic acid (BCA) protein assay reagent. The antibodies against GPR43 (Abcam, ab131003; Sigma-Aldrich, ABC299), GLUT1 (Cell Signaling, #12939), HK2 (Cell Signaling, #2867), PKM2 (Proteintech, 15822-1-AP), LDHA (Cell Signaling, #3582), P-ERK (Cell Signaling, #4370), ERK (Cell Signaling, #4695), Ac-H3 (Cell Signaling, #8173), Ac-H4 (Abcam, ab51997), G(α)i (Cell Signaling, #5290), PLCγ1 (Cell Signaling, #5690), PKC (Cell Signaling, #2056), and β-actin(Cell Signaling, #4970) were purchased from the designated manufacturers.

### Cell Apoptosis Analysis

An annexin V-FITC/PI Apoptosis Detection Kit (Vazyme, Nanjing, China #A211-01) was used according to the protocols from the manufacturer. The cells were detected using a FACSCalibur flow cytometer. The data were analyzed using FlowJo software (FLOWJO, OR, USA).

### Glycolysis Analysis

Lactate production and glucose uptake in the CRC cells were detected using the Lactate Colorimetric Assay Kits (Biovision CA, USA, #K627-100) and the Glucose Uptake Fluorometric Assay Kits (Biovision, #K666-100) according to the protocols of the manufacturer.

### The ECAR and OCR

The ECAR and OCR were measured by using the Seahorse XF Glycolysis Stress Test Kit and Cell Mito Stress Test Kit (Seahorse Bioscience, MA, USA) at the Seahorse Bioscience XF96 Extracellular Flux Analyzer according to the instructions from the manufacturer.

### The siRNA Transfection

All the siRNA were synthesized by RiboBio (Guangzhou, China). The targeting sequences of GPR43 siRNA were 5′- GGATGCCAAGTTCGGACTT-3′. The targeting sequences of negative control were 5′-UUCUCCGAACGUGUCACGUTT-3′. The siRNA targeting GPR43 was transfected into the CRC cells using Lipofectamine 2000 (Invitrogen, MA, USA) according to the instructions from the manufacturer.

### Statistical Analysis

Statistical analysis was performed with Prism 8 (GraphPad Software, CA, USA). All data were analyzed for the differences between the different treatment groups using the Student's *t*-test. The differences with *p* ≤ 0.05 were considered significant.

## Results

### BO Treatment Protects Mice From CAC

**Figure 1:**
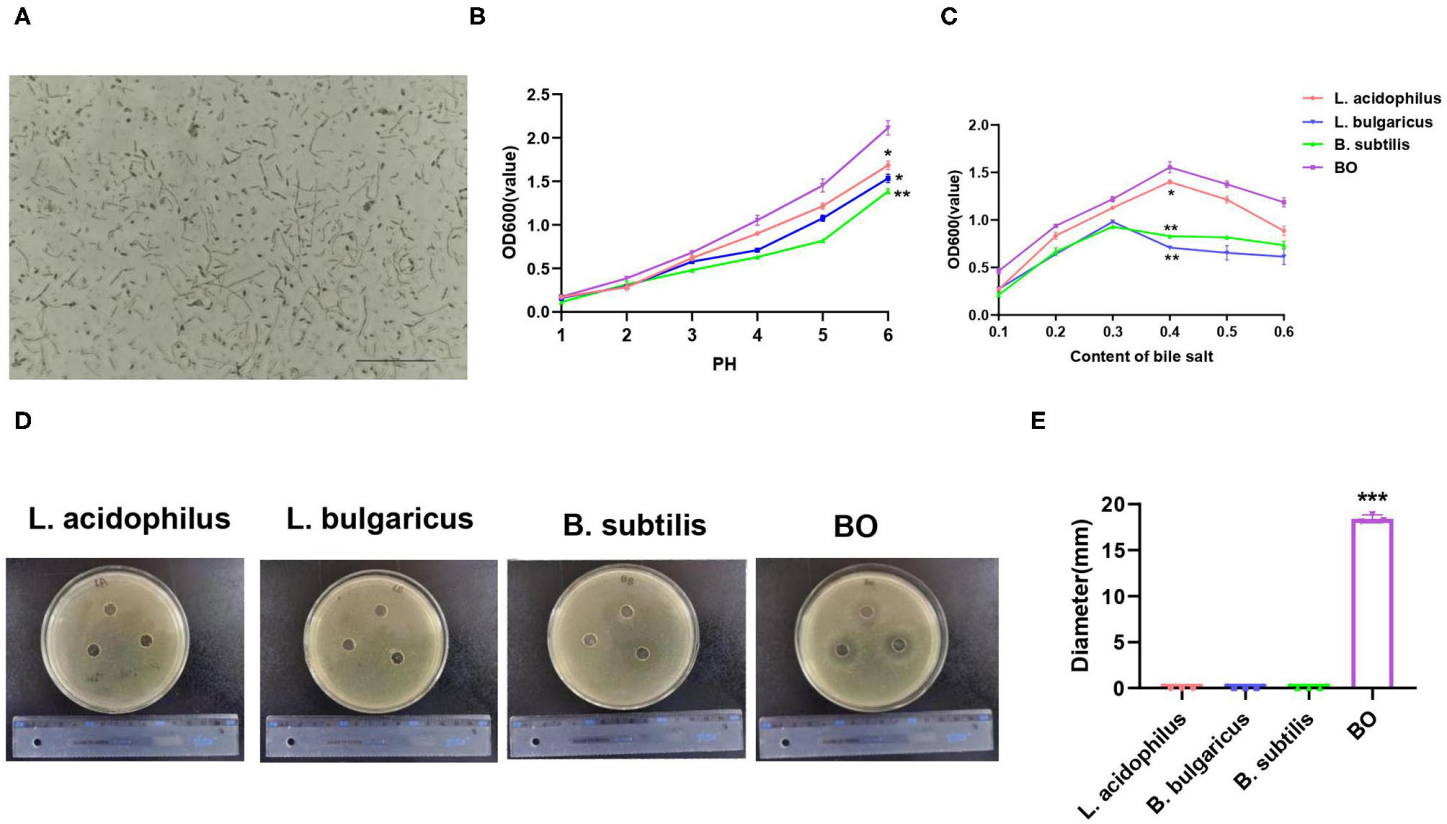
The morphological and probiotic characteristics of Bornlisy (BO). **(A)** The morphological characteristics of BO were observed under a light microscope (scale bar, 250 μm). **(B)** The single strain and fermentation mixture were inoculated in the specific liquid medium at different pH and were cultured at 37°C for 3 h at 100 r/min, and optical density (OD) 600 was measured by spectrophotometer. **(C)** The single strain and fermentation mixture were inoculated in the specific liquid medium containing different concentrations of bovine bile salt and were cultured at 37°C for 3 h at 100 r/min, and their OD 600 were measured by spectrophotometer. **(D)** *Escherichia coli* was inoculated on LB solid medium for the night at 37°C for 12 h. The representative images of the inhibition zone were shown. **(E)** The diameter of the inhibition zone was measured. Data with error bars are presented as mean ± SD. Each panel is a representative experiment of at least three independent biological replicates. *P < 0.05, **P < 0.01, and ***p < 0.001 as determined by unpaired Student's *t*-test.

In this study, the mixture of three probiotics, Bornlisy (BO), is produced by mixing *L. acidophilus, Lactobacillus bulgaricus*, and *Bacillus subtilis* on a 1:1:1 scale. The morphological characteristics of BO are shown in Figure 1A. To compare the advantages of BO over single strains, we analyzed the probiotic characteristics of BO. Both the BO and the single strain can tolerate an acidic environment with a pH above 2.0 (Figure 1B). However, BO grew faster than the single strain (Figure 1B) under the same pH condition. Compared with the single strain, the BO also showed higher growth activity in the specific medium containing bovine bile salt (Figure 1C). More importantly, BO had a stronger bacteriostatic effect on *Escherichia coli* than the single strain (Figure 1D). Therefore, we used BO with more advantages to carry out further research.

**Figure 2:**
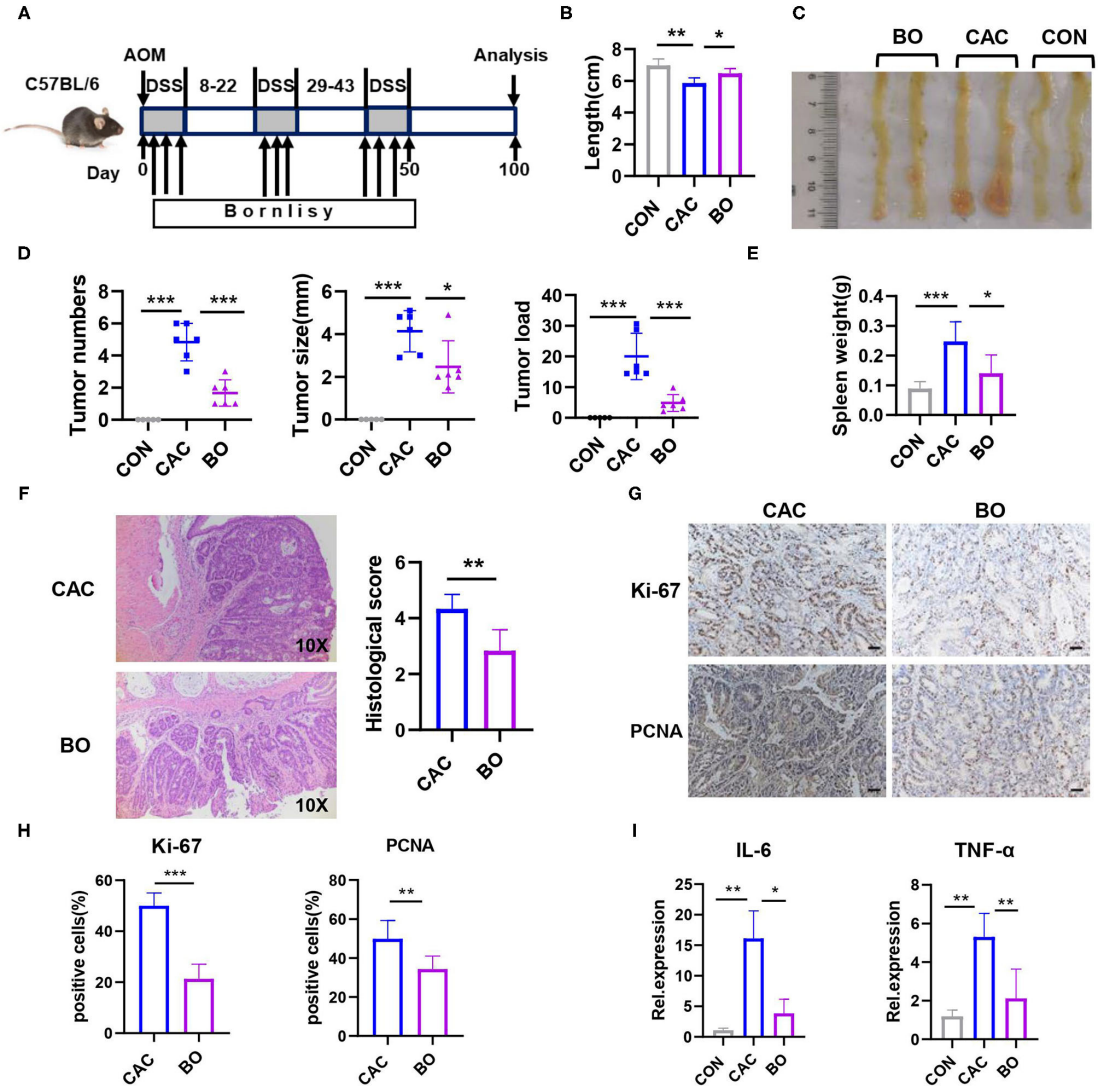
Bornlisy treatment protects the mice from colitis-associated colon cancer (CAC). **(A)** C57BL/6J mice (*n* = 5 for each group) were injected intraperitoneally with azoxymethane (AOM) (10 mg/kg) on day 1, 2% dextran sodium sulfate (DSS) was subsequently added to the drinking water for 7 consecutive days. Three cycles of DSS treatment were used. The mice were orally inoculated with BO (10 ml/kg) once every 2 days during the DSS treatment. After induction of tumorigenesis (100 days), the mice were euthanized. The spleens and colons were removed. **(B)** Colon length was measured. **(C)** The representative images of colon tumors were shown. **(D)** Tumor number, tumor size, and tumor load in the colons were measured. **(E)** The spleens from mice were weighted. **(F)** The histological analysis of colon tumors was shown by H&E staining. The histological score was assessed by a pathologist (scale bars, 50 μm). **(G)** Tumor tissues were stained for Ki-67 and proliferating cell nuclear antigen (PCNA) (scale bars, 50 μm). **(H)** The percentages of Ki-67-positive and PCNA-positive tumor cells were quantified. **(I)** mRNA expressions of *IL-6* and *TNF-*α in tumors were detected using quantitative real-time PCR (qPCR). Data with error bars are represented as mean ± SD. Each panel is a representative experiment of at least three independent biological replicates. *p < 0.05, **p < 0.01, and ***p < 0.001 as determined by unpaired Student's *t*-test.

To assess whether BO has an effect on the development of CAC, the mice were treated with azoxymethane (AOM) and dextran sodium sulfate (DSS) to induce the development of CAC, meanwhile, BO was orally inoculated one time every 2 days during the DSS treatment (Figure 2A). The mice were euthanized on the 100th day, and the colons and tumors were evaluated. As shown in Figure 1B, the BO-treated mice had longer colons as compared with the CAC mice (Figure 2B). Moreover, the BO treatment inhibited tumor numbers, tumor sizes, and tumor loads (Figures 2C,D). The spleens were significantly shrunken in the BO-treated mice as compared with the CAC mice (Figure 2E). The histological score of tumor tissues was significantly lower in the BO-treated mice than in the CAC mice (Figure 2F). An immunohistochemical (IHC) analysis was used to detect cellular proliferation in colonic tumors. The tumors from the BO-treated group had decreased the expression of proliferating cell nuclear antigen (PCNA) and Ki-67, compared with those from the CAC group (Figures 2G,H). The mRNA levels of pro-inflammatory genes were also examined. The BO-treatment significantly inhibited the mRNA expression of *IL-6* and *TNF-*α in tumor tissues (Figure 2I). Taken together, our results indicate that BO exerts protective effects against the CAC.

### BO Inhibits Proliferation and Metastasis of CRC cells *in vitro*

**Figure 3:**
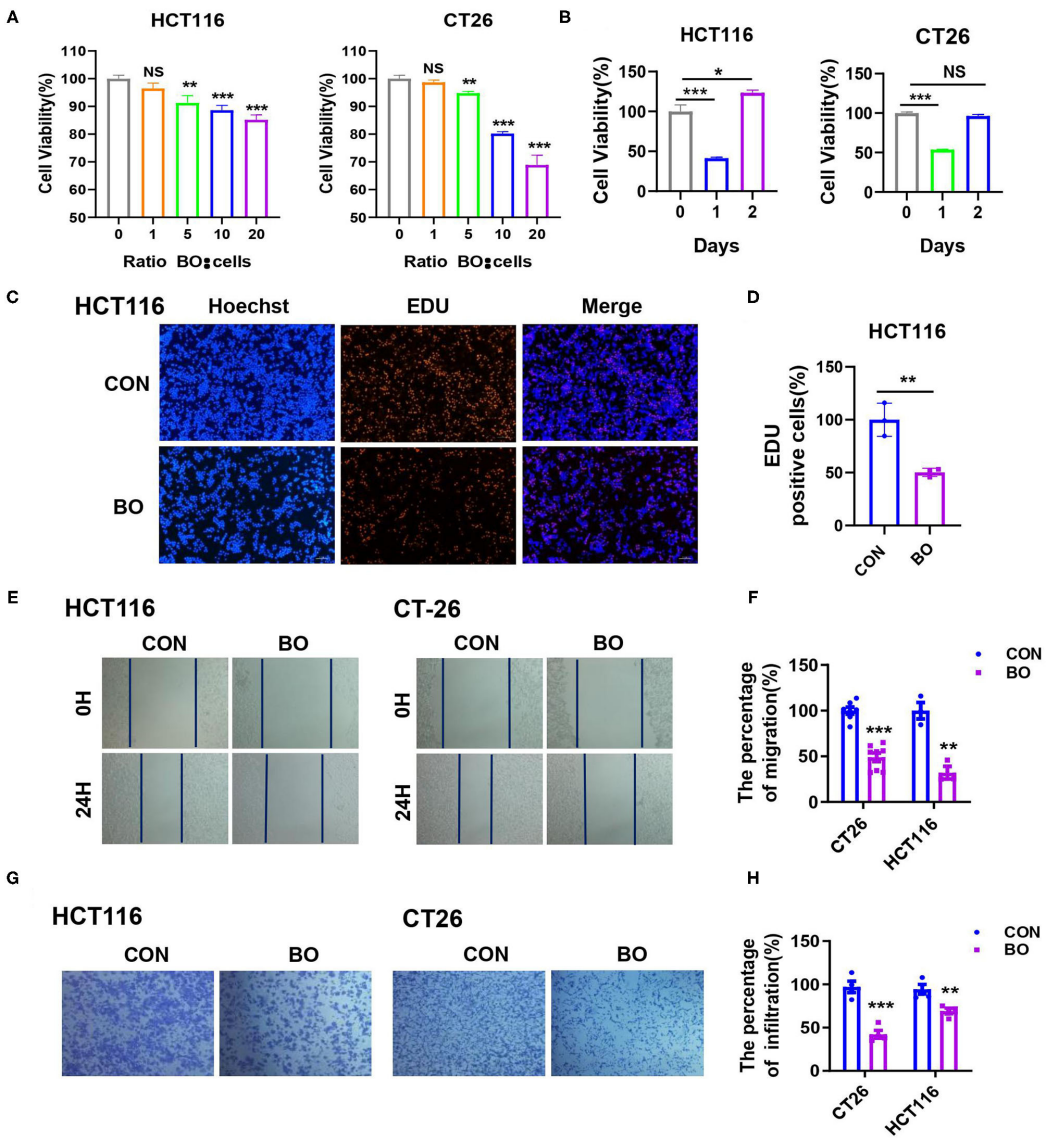
Bornlisy inhibits proliferation and metastasis of colorectal cancer (CRC) cells *in vitro*. **(A)** The HCT116 and CT26 cells were treated with BO of different concentrations for 24 h. The cell proliferation was measured by CCK8 assay. **(B)** The HCT116 and CT26 cells were treated with BO (multiplicity of infection [MOI] = 5) for different days. The cell proliferation was determined by the CCK-8 assay. **(C,D)** The HCT116 cells were treated with BO (MOI = 5, 24 h). The cell proliferation was evaluated using immunofluorescence staining. The EdU-positive cells were calculated (scale bar, 100 μm). **(E,F)** The HCT116 cells and CT26 cells were treated with BO (MOI = 5, 24 h). The migratory ability was detected by wound healing assays. The percentage of migration was measured. **(G,H)** The HCT116 cells and CT26 cells were treated with BO (MOI = 5, 24 h). The infiltration ability was detected by a transwell assay. The percentage of infiltration was measured. Data with error bars are presented as mean ± SD. Each panel is a representative experiment of at least three independent biological replicates. *P < 0.05, **P < 0.01, and ***p < 0.001 as determined by unpaired Student's *t*-test.

To investigate the effect of BO on the proliferation of CRC cells *in vitro*, cell proliferation was detected using Cell Counting Kit-8 (CCK8; Dojindo Laboratories, Japan) and 5-Ethynyl-2′-deoxyuridine (EdU). BO significantly inhibited the proliferation of HCT116 and CT26 cells in both the dose-dependent manner (Figure 3A) and the time-dependent manner (Figure 3B). The minimum effective concentration is a multiplicity of infection (MOI) = 5, while the best inhibitory time is 24 h. In the EdU assay, fewer cells were found in the BO-treated cells than in the control group (Figures 3C,D; Supplementary Figure 1). The effect of BO treatment on cell invasion was further investigated. By using the scratch test and the transwell migration assay, we found that the migration and metastasis ability of the CRC cells were significantly inhibited upon the BO treatment (Figures 3E–H). Together, these results suggested that the BO inhibits tumorigenesis both *in vivo* and *in vitro*.

### BO Inhibits Glucose Metabolism in Cancer Cells

**Figure 4:**
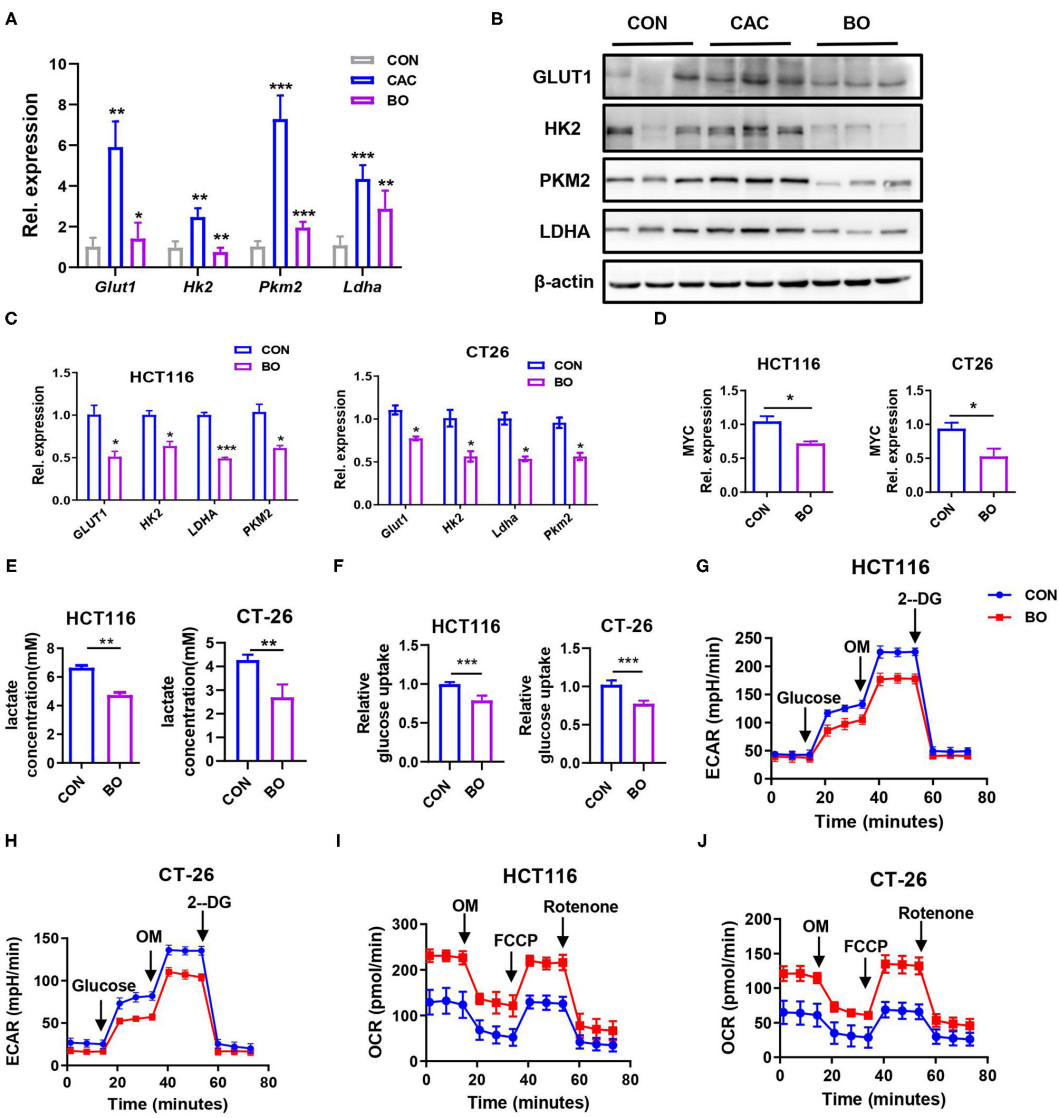
Bornlisy inhibited aerobic glycolysis of colon cancer cells *in vivo* and *in vitro*. **(A)** The mice were treated as described in Figure 1A. The tumor tissues were separated. The mRNA expressions of *PKM2, PFKL, LDHA, HK2, GLUT1, PGK1*, and *PGAM1* in tumors tissues were detected by qPCR. **(B)** The protein levels of GLUT1, HK2, PKM2, and LDHA in tumors tissues were detected by western blots. **(C)** The HCT116 cells and CT26 cells were treated with BO for 24 h (MOI = 5). The mRNA expressions of *GLUT1, HK2, PKM2, LDHA*, and *PFKL* in the HCT116 cells and CT26 cells were detected by qPCR. **(D)** The HCT116 cells and CT26 cells were treated with BO for 24 h (MOI = 5). The mRNA expressions of *MYC* in HCT116 cells and CT26 cells were detected by qPCR. **(E,F)** The HCT116 cells and CT26 cells were treated with BO (MOI = 5, 24 h). The cell supernatant is collected. Lactate production was detected by Lactate Colorimetric Assay Kits. Glucose uptake was detected by Glucose Uptake Fluorometric Assay Kits. **(G,H)** The HCT116 cells and CT26 cells were treated with BO (MOI = 5, 24 h). The extracellular acidification rate (ECAR) of HCT116 cells and CT26 cells were measured by the Seahorse XF Glycolysis Stress Test Kit. **(I,J)** The HCT116 cells and CT26 cells were treated with BO (MOI = 5, 24 h). The oxygen consumption rate (OCR) of HCT116 cells and CT26 cells were measured by the Cell Mito Stress Test Kit. Data with error bars are presented as mean ± SD. Each panel is a representative experiment of at least three independent biological replicates. *P < 0.05, **P < 0.01, ***p < 0.001 as determined by unpaired Student's *t*-test.

The metabolic reprogramming, especially the glucose metabolism to hypoxic glycolysis in the tumor environment has been reported in many studies. Therefore, we examined several key enzymes in glucose metabolism in our CAC mice. The mRNA expression of *Glut1, Hk2, Ldha*, and *Pkm2* were downregulated in the tumor tissues of BO-treated CAC mice (Figure 4A). Similar results were found in the protein levels of these enzymes (Figure 4B). Consistently, BO also inhibited the expression of these enzymes in the HCT116 and CT26 cells (Figure 4C). Oncogenes, such as MYC, upregulate glycolysis activity (51). We also found that the expression of MYC was downregulated after the BO treatment (Figure 4D). To further verify the effect of BO on glucose metabolism, glucose uptake, and lactic acid production were measured. We found that BO decreased glucose uptake and lactic acid production in the HCT116 and CT26 cells (Figures 4E,F). The extracellular acidification rate (ECAR) was decreased while the oxygen consumption rate (OCR) was increased in the BO-treated cancer cells (Figures 4G–J). In summary, our results show that BO inhibits glycolysis of the CRC cells.

### BO Inhibits Colon Cancer Cell Proliferation by Reducing Glycolysis

**Figure 5:**
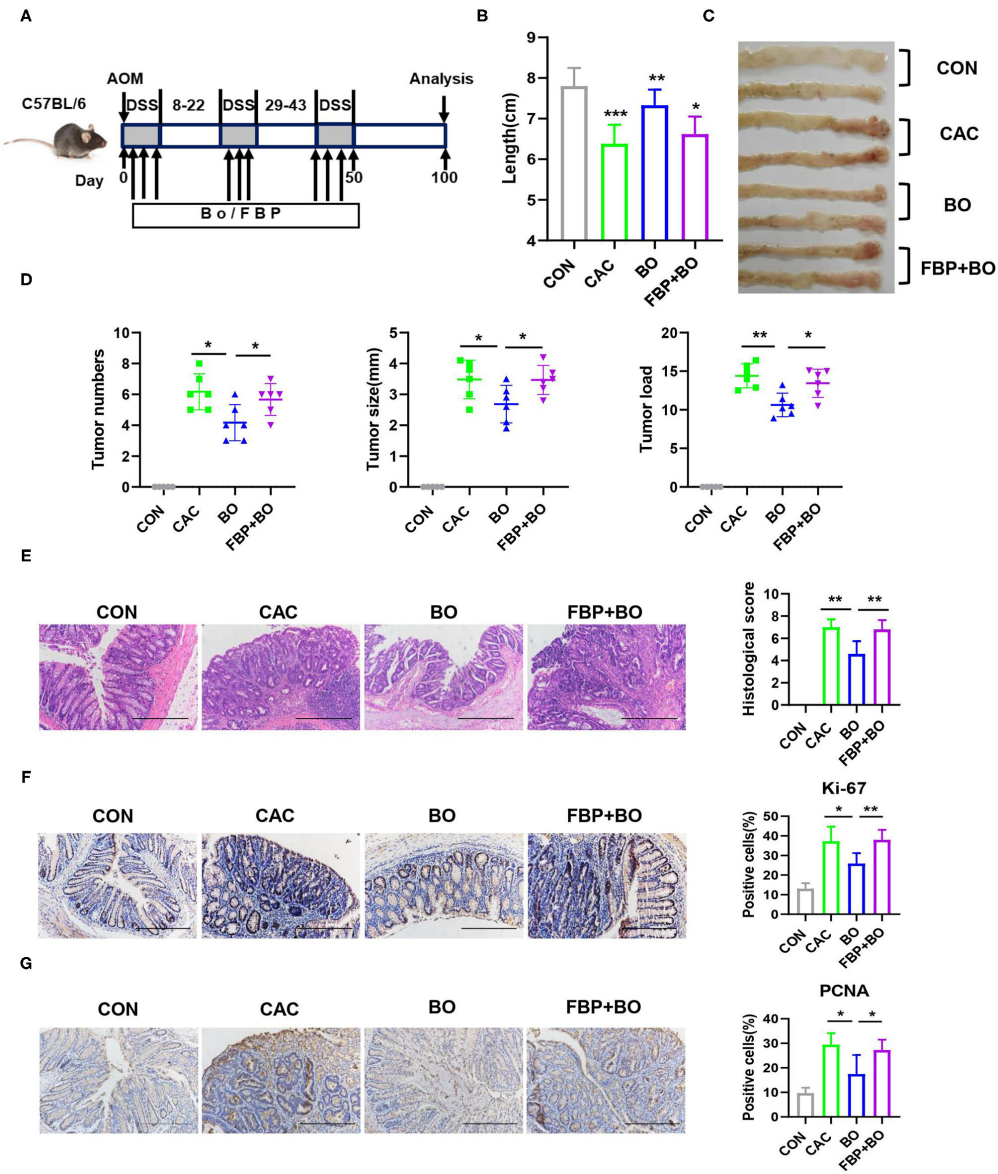
Bornlisy inhibits colon cancer cell proliferation by reducing glycolysis. **(A)** The C57BL/6J mice (*n* = 5 for each group) were intraperitoneally treated with fructose-2,6-biphosphate (FBP) (500 mg/kg, once every 2 days) and orally treated with BO (10 ml/kg, once every 2 days) during AOM-DSS administration. After induction of tumorigenesis (100 days), the mice were euthanized and the colons were removed. **(B)** The representative images of colons were shown, and the colons' lengths were measured. **(C)** The representative images of colon tumors were shown. **(D)** Tumor number, tumor size, and tumor load in colons were measured. **(E)** The histological colon tumor images using H&E staining were shown. The histological score was assessed by a pathologist (scale bars, 500 μm). **(F,G)** The tumor tissues were stained for Ki-67 and PCNA (scale bar: 500 μm). The percentages of Ki-67-positive and PCNA-positive cells were quantified. Data with error bars are represented as mean ± SD. Each panel is a representative experiment of at least three independent biological replicates. Scale bars, 50 mm. *p < 0.05, **p < 0.01, and ***p < 0.001 as determined by unpaired Student's *t*-test.

To identify the role of aerobic glycolysis in the development of CAC, fructose-2,6-biphosphate (FBP), a glycolytic activator, was administrated to the C57BL/6J mice during the AOM-DSS treatment (Figure 5A). As described above, the BO-treated mice had longer colons and decreased tumor loads compared with the PBS-treated control mice. However, FBP administration with BO reversed the protective role of BO, presenting as shorter colons and increased tumor numbers, tumor size, and tumor loads (Figures 5B–D). Moreover, the severity of proliferation in the colon was also reversed in the BO-treated mice after FBP administration (Figure 5E). Similar results were found using the PCNA and Ki-67 IHC staining (Figures 5F,G). These results together suggested that the BO inhibits colon cancer cell proliferation through downregulating glycolysis in the cancer cells.

### GPR43 Is a Targeted Receptor of BO in Suppressing CRC Proliferation

**Figure 6:**
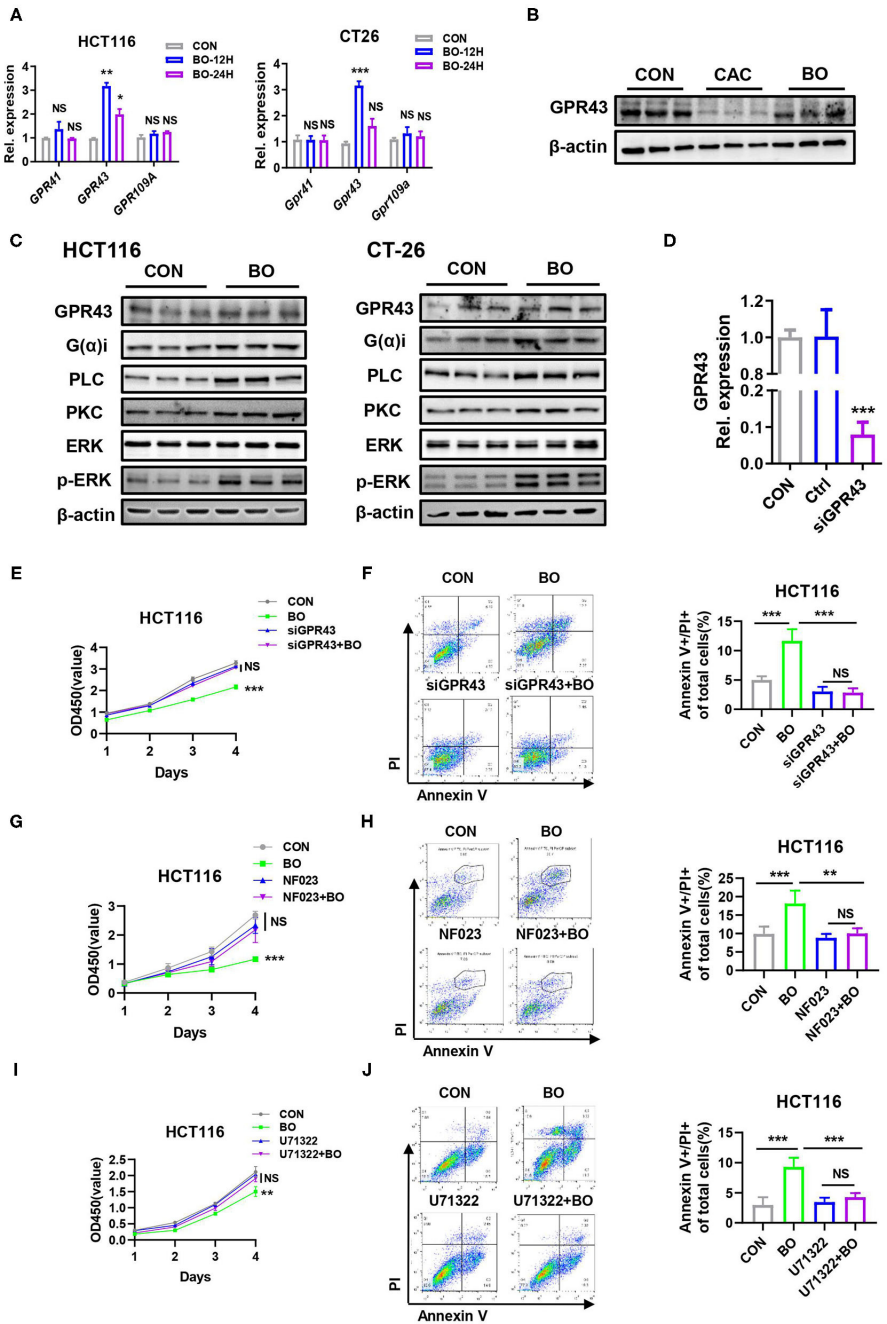
A GPR43 is a targeted receptor of BO in suppressing CRC proliferation. **(A)** The HCT116 cells and CT26 cells were treated with BO for 12 and 24 h (MOI = 5). The mRNA expressions of *GPR41, GPR43*, and *GPR109A* in the HCT116 cells and CT26 cells were detected by qPCR. **(B)** The mice were treated as described in Figure 1A. The tumor tissues were separated. The protein levels of GPR43 in tumors tissues were detected by the western blots. **(C)** The HCT116 cells and CT26 cells were treated with BO for 12 and 24 h (MOI = 5). The protein levels of GPR43, G(α)i, PLC, PKC, p-ERK, and total ERK in the HCT116 cells and CT26 cells were detected by western blots. **(D)** The HCT116 cells were treated with siGPR43 duplexes for 48 h. The efficiency of GPR43 knockdown in HCT116 cells was measured by qPCR. **(E,F)** The HCT116 cells were treated with siGPR43 duplexes for 48 h and then treated with BO (MOI = 5, 24 h). The cell proliferation was measured by a CCK8 assay. The cell apoptosis was monitored by flow cytometry. The percentage of the apoptotic cell (Annexin V^+^/PI^+^) was calculated. **(G,H)** The HCT116 cells were stimulated with BO (MOI = 5, 24 h) after pretreatment with NF023 (10 mM) for 4 h. The cell proliferation was measured by a CCK8 assay. The cell apoptosis was monitored by flow cytometry. The percentage of an apoptotic cell (Annexin V^+^/PI^+^) was calculated. **(I,J)** The HCT116 cells were stimulated with BO (MOI = 5, 24 h) after pretreatment with U73122 (1 mM) for 4 h. The cell proliferation was measured by a CCK8 assay. The cell apoptosis was monitored by flow cytometry. The percentage of the apoptotic cell (Annexin V^+^/PI^+^) was calculated. Data with error bars are presented as mean ± SD. Each panel is a representative experiment of at least three independent biological replicates. *P < 0.05, **P < 0.01, and ***p < 0.001 as determined by unpaired Student's *t*-test.

Some Gram-positive bacteria exert anti-inflammatory effects through the toll-like receptor (TLR) signaling pathway (52, 53), which prompts us to investigate whether the TLR signaling was activated in the BO-treated CRC cells. However, no difference in the transcriptional level of *TLR1, TLR2, TLR4, and TLR6* was found in the CRC cells upon BO stimulation (Supplementary Figure 2A). Next, we examined the role of histone deacetylase butyrate (HDAC) inhibition in enhancing antibacterial activity (54). However, the amount of acetylated H3 and H4 showed no difference upon BO treatment (Supplementary Figure 2B). The SCFAs activate multiple signaling pathways by binding to GPR41, GPR43, and GPR109A with varying affinities (55). GPR43 recognizes SCFAs and is involved in the inhibition of colorectal cancer (56). As shown in Figure 5A, the BO-treatment upregulated the mRNA expression of GPR43 but had no significant effect on the mRNA expressions of GPR41 and GPR109a (Figure 6A). The protein level of GPR43 in the tumor tissues was also upregulated (Figure 6B). GPR43 couples to Gαi proteins, which results in the activation of phospholipase C (PLC), protein kinase C (PKC), and the following extracellular signal-regulated kinase (ERK) (57). The BO treatment significantly activated this GPR43-Ga(i/o)-PLC-PKC-ERK signaling pathway in HCT116 and CT26 cells (Figure 6C). To determine whether the inhibition of BO on the proliferation of cancer cells is mediated by GPR43, GPR43 was knocked down by using siGPR43. The transfection efficiency of siGPR43 is shown in Figure 6D. The CCK8 assays suggested that GPR43 knockdown combined with BO offset the inhibitory effect of BO on cell proliferation (Figure 6E). Moreover, annexin/PI staining showed that GPR43 knockdown combined with BO eliminated the inhibiting effect of BO on cell proliferation (Figure 6F). Similar results were found by using Ga(i/o) blocker (NF023) (Figures 6G,H), PLC inhibitor (U73122) (Figures 6I,J), and PKC inhibitor (Go6983) (Supplementary Figures 2C,D). These results together suggest that BO inhibited tumor proliferation through activating GPR43- Ga(i/o)-PLC–PKC–ERK signaling pathway.

### Activating GPR43 Inhibits Aerobic Glycolysis in the Cancer Cells

**Figure 7:**
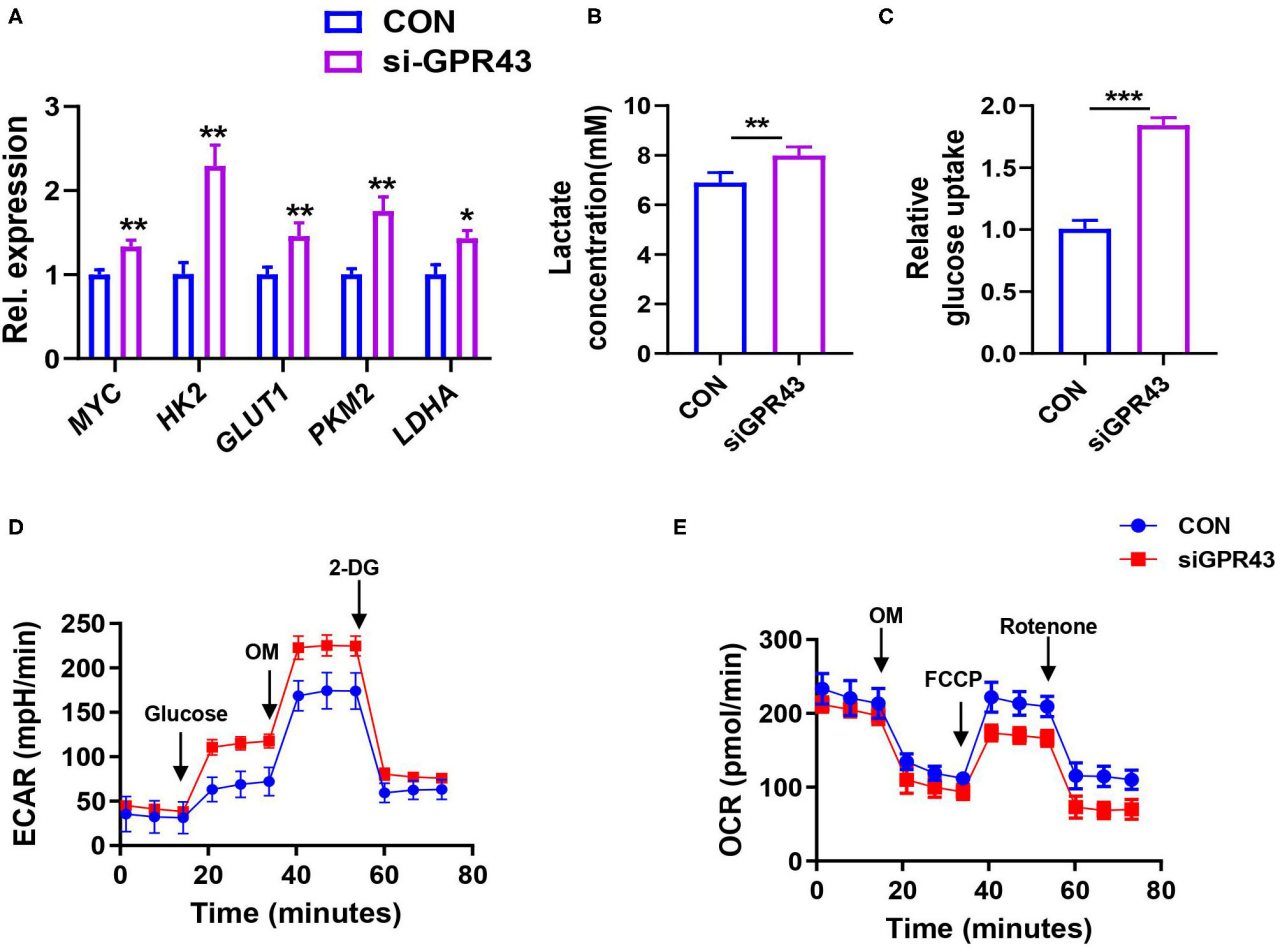
Activating GPR43 can inhibit aerobic glycolysis in the CRC cells. The HCT116 cells were treated with siGPR43 duplexes for 48 h. **(A)** The mRNA expressions of *MYC, GLUT1, HK2, PKM2*, and *LDHA* in the HCT116 cells were detected by qPCR. **(B)** Lactate production of HCT116 cells was detected by Lactate Colorimetric Assay Kits. **(C)** Glucose uptake of HCT116 cells was detected by Glucose Uptake Fluorometric Assay Kits. **(D)** The ECAR of HCT116 cells was measured by the Seahorse XF Glycolysis Stress Test Kit. **(E)** The OCR of HCT116 cells was measured by the Cell Mito Stress Test Kit. Data with error bars are presented as mean ± SD. Each panel is a representative experiment of at least three independent biological replicates. *P < 0.05, **P < 0.01, and ***p < 0.001 as determined by unpaired Student's *t*-test.

We further examined whether GPR43 regulates glycolysis in cancer cells. We found that GPR43 knockdown upregulated the expression of the key enzymes in the glucose metabolism, including MYC, GLUT1, HK2, LDHA, and PKM2 (Figure 7A). GPR43 also led to increased lactate production, increased glucose uptake, increased ECAR, and decreased OCR in HCT116 cells (Figures 7B–E). Our data suggested that GPR43 inhibits aerobic glycolysis in the CRC cells.

## Discussion

**Figure 8:**
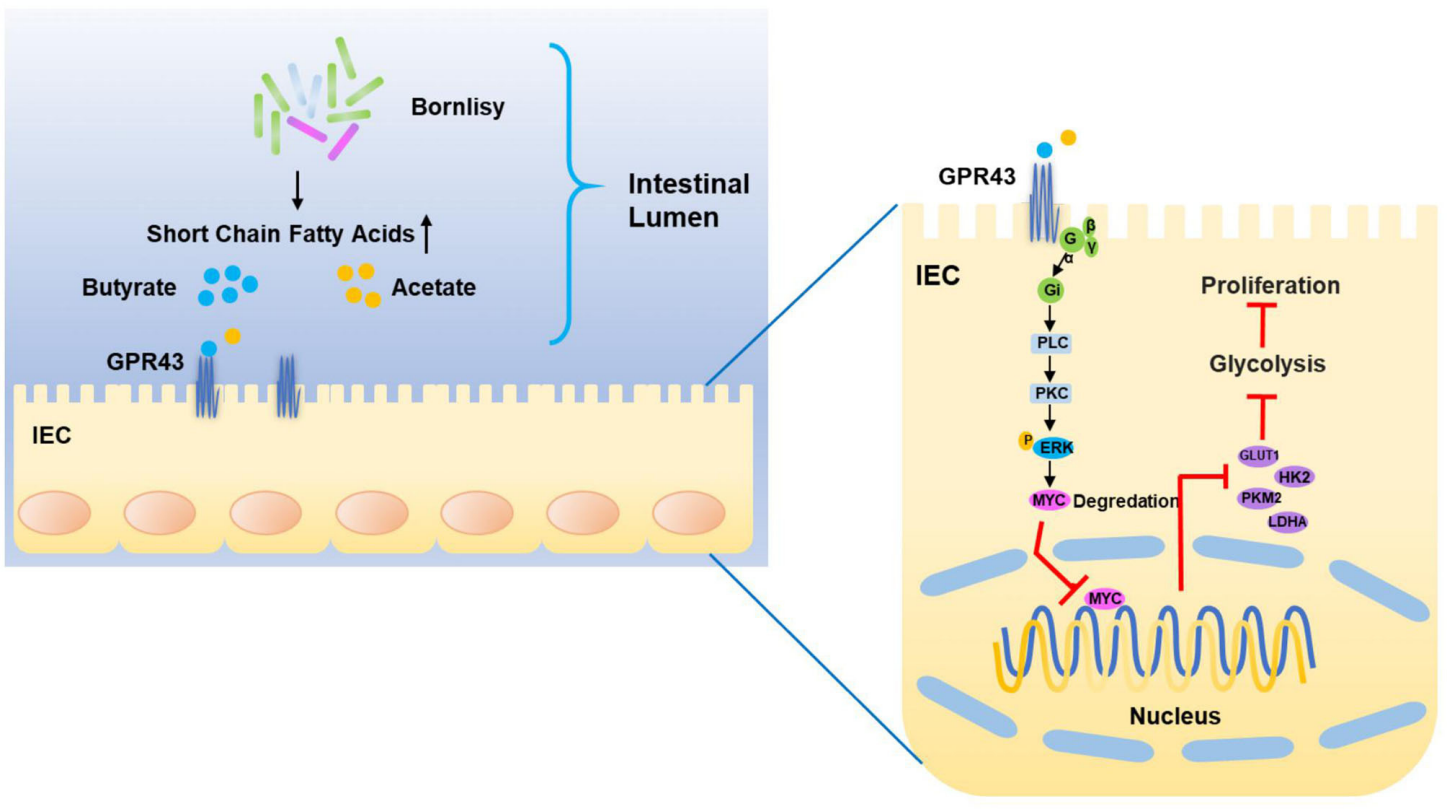
Proposed model of the mechanism underlying the BO-induced suppression of CAC *via* inhibiting the GPR43-mediated glycolysis.

More and more evidence suggests that probiotics exhibit extensive antitumor effects in different kinds of cancers (58–60). However, the mechanism by which the probiotics represses the CRC has still not been well-studied. Based on our research, we found that the probiotics repress aerobic glycolysis to repress the malignant progression of CRC. Furthermore, our studies *in vivo* and *in vitro* revealed that the development of CRC could be repressed by the probiotics significantly. Moreover, our research revealed that GPR43 performed an important role *via* function analysis in the CRC cells in the process of tumor suppression, and was an important targeting receptor of probiotics. We deduced that the probiotics negatively regulate the tumorigenesis and the metabolic process of glucose *via* activating GPR43 in CRC (Figure 8).

Previous studies have demonstrated that the probiotics were able to prevent tumorigenesis and progression after being administered sufficient doses (61, 62). In this research, we proved that BO represses the proliferation of CRC cells *in vivo* and *in vitro*. The BO-treated mice had fewer tumor numbers, reduced tumor burden, and decreased tumor sizes in the CAC mouse model. *In vitro*, BO exhibits anti-proliferative and pro-apoptotic effects in the CRC cells. Normally, the cancer cells metabolize abnormally due to metabolic reprogramming. The most noticeable metabolic reprogramming existing in cancer leads to the aerobic glycolysis instead of the oxidative phosphorylation reaction in mitochondrial, which releases constant nutrients and energy utilized for the out-of-control proliferation, as is defined as the Warburg effect (32, 63, 64). Whether probiotics can regulate glucose metabolism in colon cancer is unclear. Our data demonstrated that BO administration decreased the expression of MYC and downstream glycolytic genes of GLUT1, LDHA, HK2, and PKM2 in the CRC cells, which lessened the activity of glycolysis. In consideration of the crucial performance of BO in the process of glucose metabolism in CRC, we further studied the potential underlying mechanism and downstream effectors.

Recently, it is reported that GPR43, a G protein-coupled receptor, can be activated by the SCFAs (65). FFAR2(GPR43) is downregulated in human colon cancers that matched the adjacent healthy tissue. In accordance with this, Ffar2^−/−^ mice are hyper susceptible to the development of intestinal carcinogenesis (66). *C. butyricum* reduced the contents of fecal secondary bile acids (BA), heighten the quantities of cecal SCFA, and activated the G-protein coupled receptors (GPRs), such as GPR43 and GPR109A (26). However, whether BO can activate GPCR in the intestinal epithelial cell still is unclear. To explore the targeting receptor of BO that mediates the repression of CRC cell proliferation, we examine the expression of TLR, GPCR, and acetyl-histone. Interestingly, the results suggested that BO activates the G protein-coupled receptor and that GPR43 was dramatically upregulated. It is reported that GPR43 links to either Gi/o or Gq as another subunit of heterotrimeric G protein (67). Our study showed that the BO-activated GPR43 couples to Gi/o and followed by PLC/PKC/ERK axis. To our knowledge, GPR43 negatively modulates the proliferation of CRC cells, and functions as a tumor suppressor in CRC. Consistent with the previous results, our data suggested that BO was linked with GPR43 knockdown *in vitro*, the repressive effect of BO on CRC was abrogated, exhibiting that GPR43 was a significant downstream target receptor of BO that regulated the repression of CRC cell proliferation.

The biological function of GPR43 has seldom been studied in the field of glucose metabolism; consequently, it is innovative that our findings proved that GPR43 is a negative regulator of glucose metabolism in CRC. But how GPR43 regulates glycolysis is not clear. Our results showed that BO administration enhanced the phosphorylation level of ERK (Thr202/Tyr204) and decreased the expression of MYC in the CRC cells and tumor tissues. According to the previous reports, CD47 interacted with ENO1 and protected it from degradation mediated by ubiquitin, subsequently enhancing the glycolytic activity and phosphorylation of ERK in the CRC cells (68). A LncRNA LINRIS bound to a ubiquitination site of insulin-like growth factor 2 mRNA-binding protein 2 (IGF2BP2), and this binding impeded the degradation of IGF2BP2 *via* the ubiquitination-autophagic pathway. As a typical target of IGF2BP2, downstream mRNAs of MYC mRNA, including MYC mRNA, were studied (69). These studies perhaps had suggested that the phosphorylation of ERK and the ubiquitination of MYC have a delicate relationship in glycolytic metabolism. The mechanism by which GPR43 functions in the process of glycolysis is waiting for further exploration. We will concentrate on these problems in our future studies.

## Data Availability Statement

The raw data supporting the conclusions of this article will be made available by the authors, without undue reservation.

## Ethics Statement

The animal study was reviewed and approved by the Institutional Animal Care and Use Committee at Nanjing University.

## Author Contributions

TW: study concept. TW and XL: study design and preparing the manuscript. TW, XL, SQ, CP, ZX, JQ, SZ, YH, and PC: data acquisition. TW, PC, SZ, and XL: data analysis and interpretation. XL: statistical analysis. All authors contributed to the article and approved the submitted version.

## Funding

This work was supported by grants from the National Natural Science Foundation of China (81772542, 82072648, and 81872114), the Natural Science Foundation of Jiangsu Province (BK20211508 and BK20190134), the Fundamental Research Funds for the Central Universities (021414380472), and the Jiangsu Provincial Special Program of Medical Science (BE2019617).

## Conflict of Interest

The authors declare that the research was conducted in the absence of any commercial or financial relationships that could be construed as a potential conflict of interest.

## Publisher's Note

All claims expressed in this article are solely those of the authors and do not necessarily represent those of their affiliated organizations, or those of the publisher, the editors and the reviewers. Any product that may be evaluated in this article, or claim that may be made by its manufacturer, is not guaranteed or endorsed by the publisher.
