# Supplementary material for: Bornlisy Attenuates Colitis-Associated Colorectal Cancer via Inhibiting GPR43-Mediated Glycolysis
Source: Front Nutr. 2021 Nov 12;8:706382. doi: 10.3389/fnut.2021.706382 (PMC8636091; doi:10.3389/fnut.2021.706382)
Supplement: Supplementary file 1 [file Presentation_1.PPTX]

## Slide 1
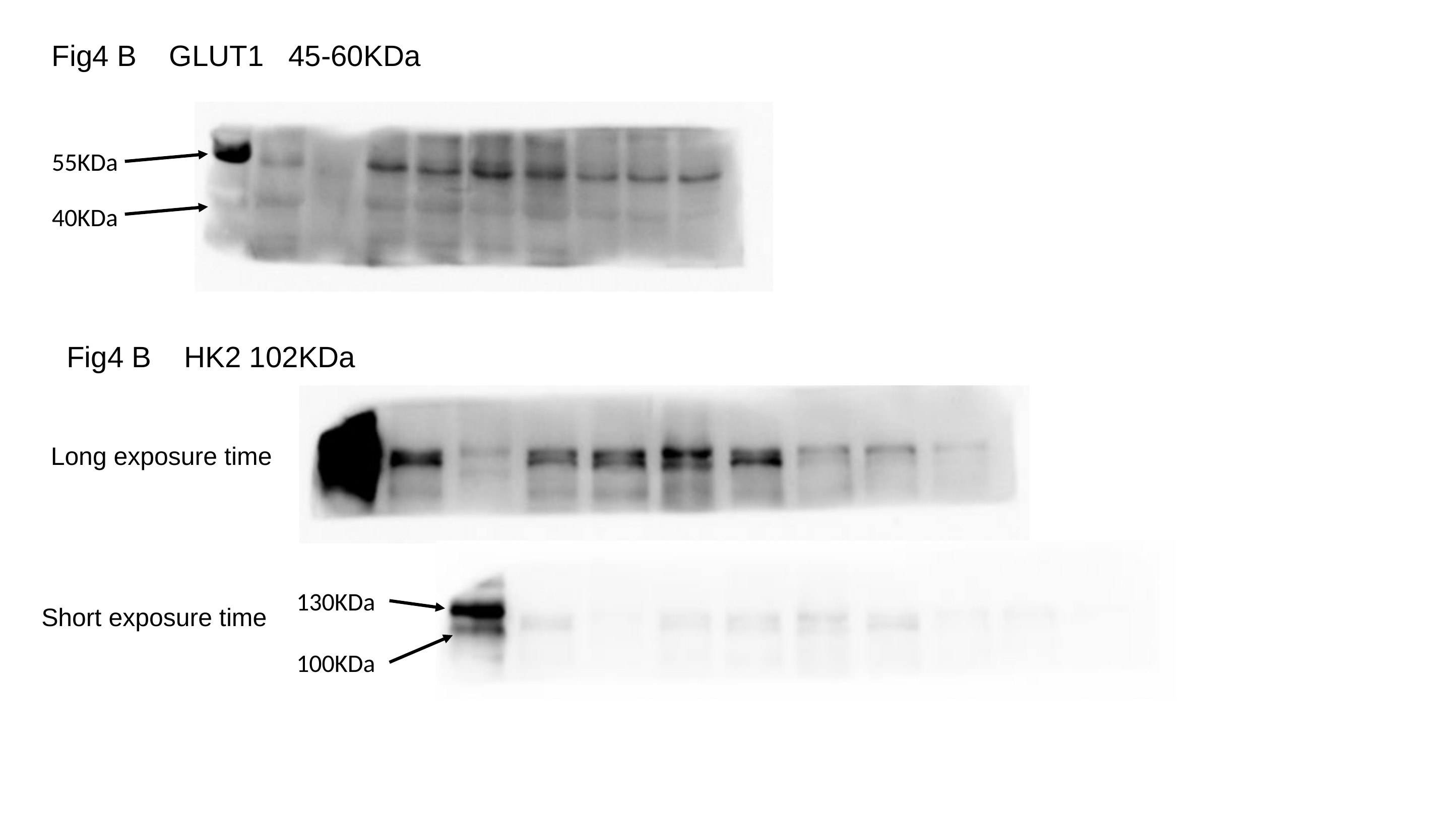

Fig4 B GLUT1 45-60KDa
55KDa
40KDa
Fig4 B HK2 102KDa
Long exposure time
130KDa
100KDa
Short exposure time

## Slide 2
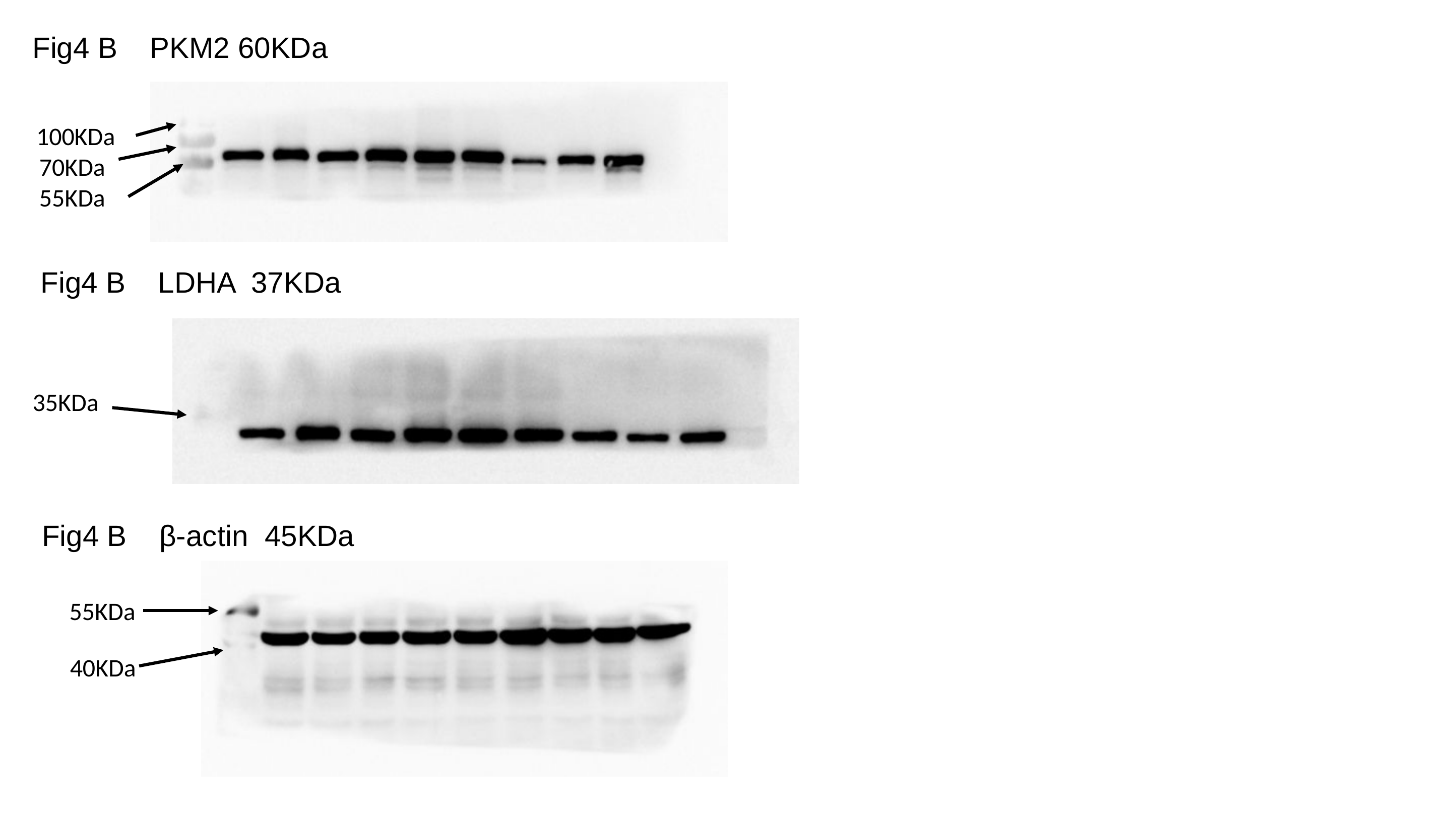

Fig4 B PKM2 60KDa
100KDa
70KDa
55KDa
Fig4 B LDHA 37KDa
35KDa
Fig4 B β-actin 45KDa
55KDa
40KDa

## Slide 3
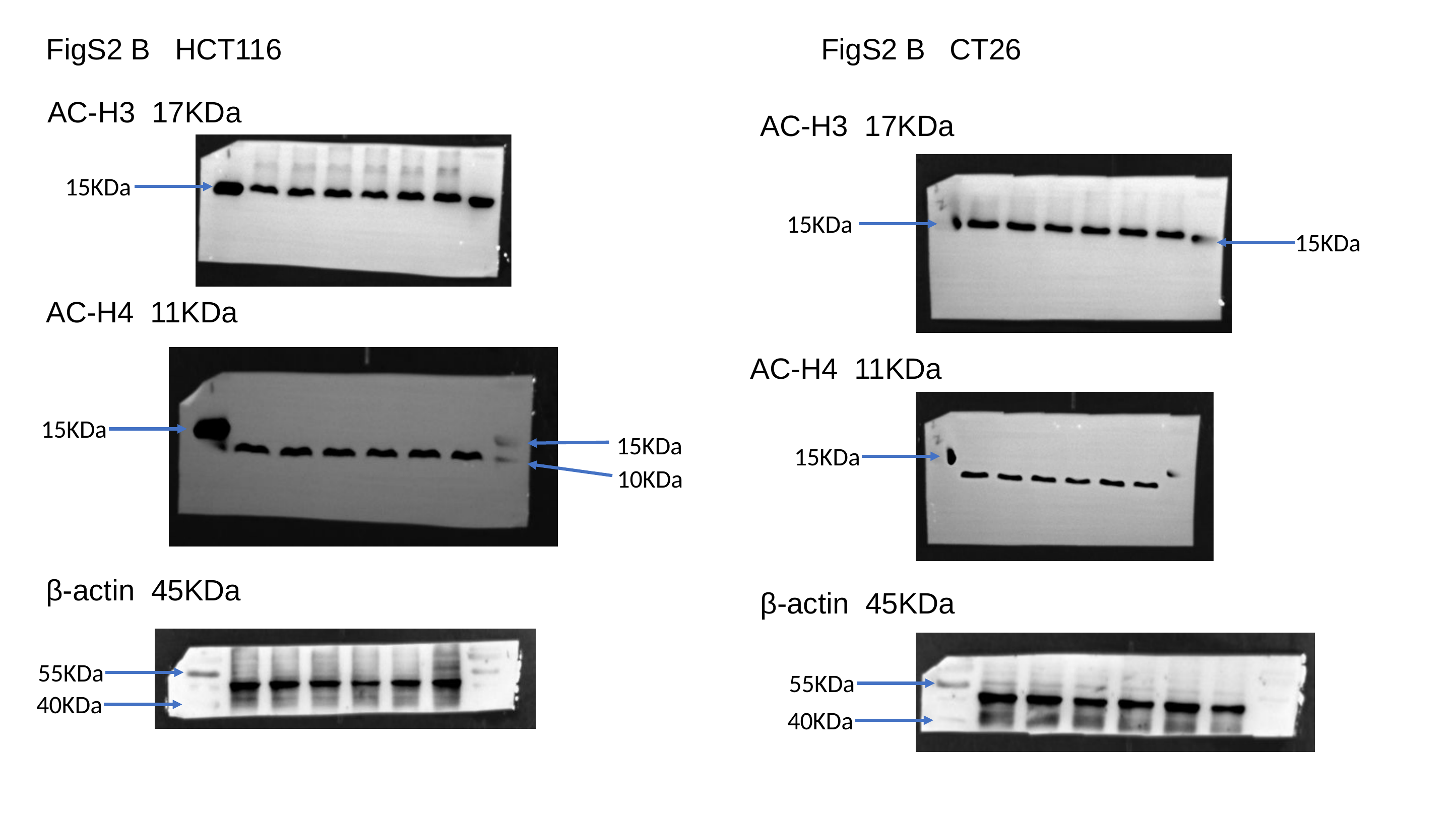

FigS2 B HCT116
FigS2 B CT26
AC-H3 17KDa
AC-H3 17KDa
15KDa
15KDa
15KDa
AC-H4 11KDa
AC-H4 11KDa
15KDa
15KDa
15KDa
10KDa
β-actin 45KDa
β-actin 45KDa
55KDa
55KDa
40KDa
40KDa

## Slide 4
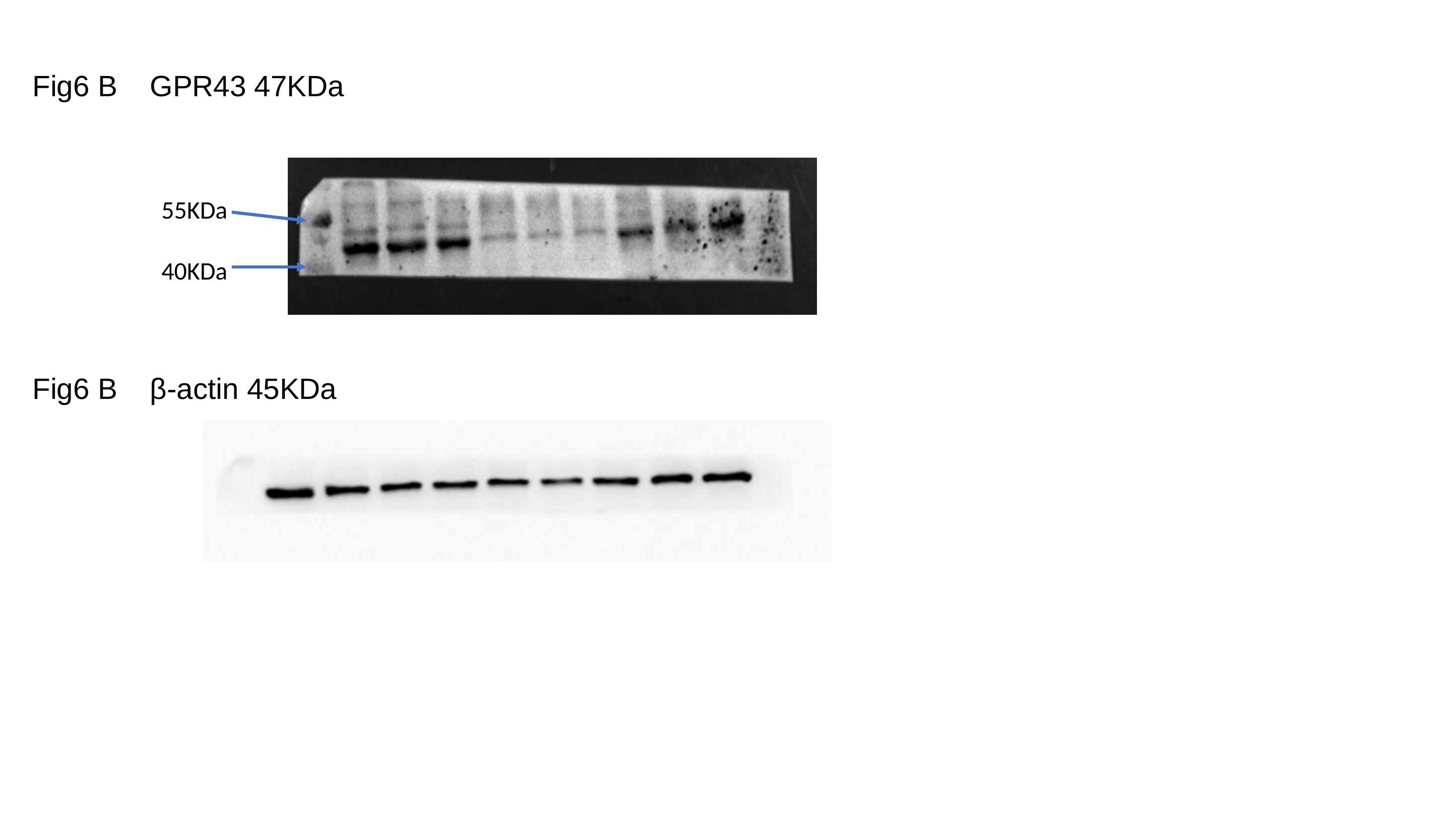

Fig6 B GPR43 47KDa
55KDa
40KDa
Fig6 B β-actin 45KDa

## Slide 5
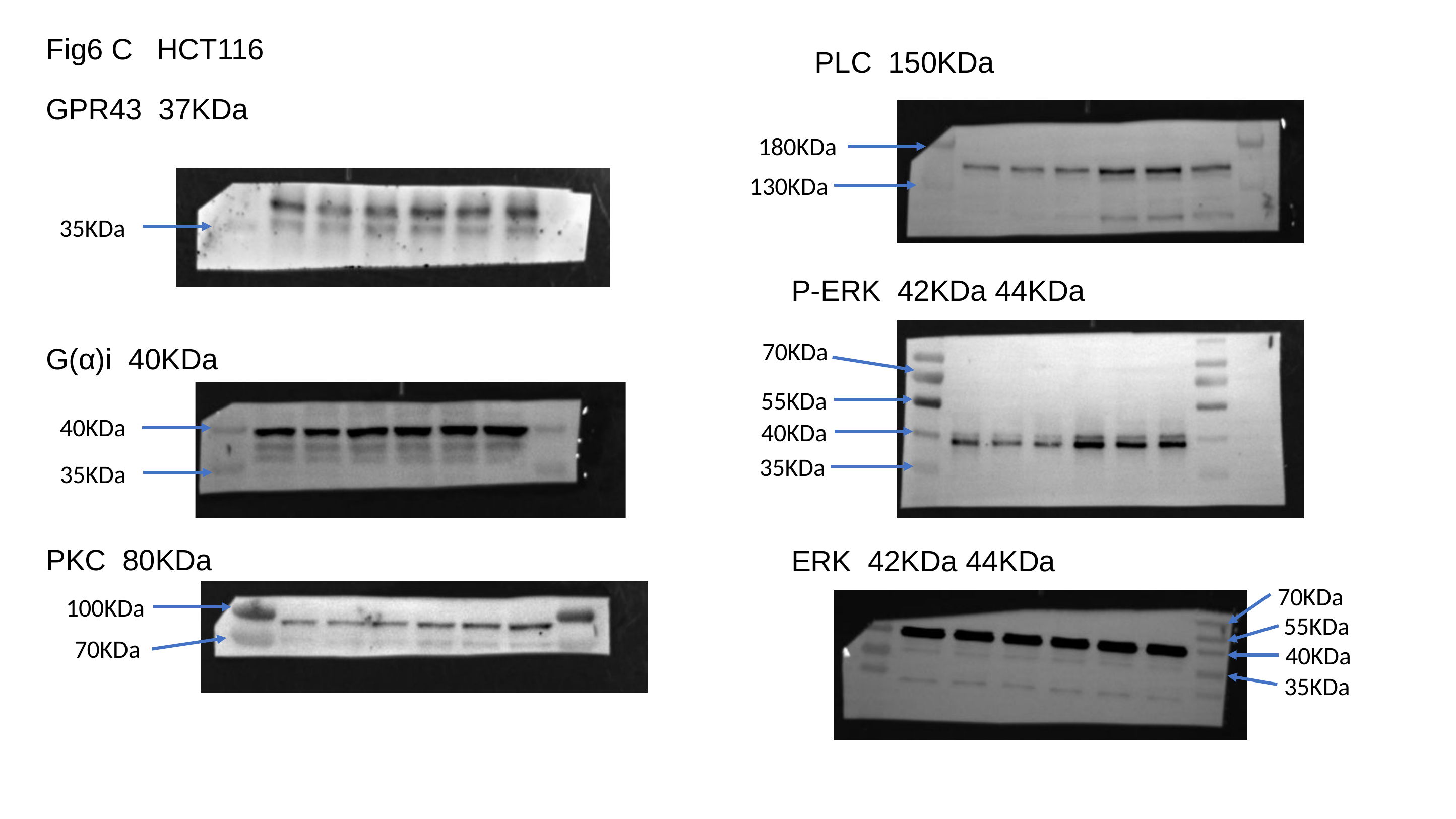

Fig6 C HCT116
PLC 150KDa
GPR43 37KDa
180KDa
130KDa
35KDa
P-ERK 42KDa 44KDa
70KDa
G(α)i 40KDa
55KDa
40KDa
40KDa
35KDa
35KDa
PKC 80KDa
ERK 42KDa 44KDa
70KDa
100KDa
55KDa
70KDa
40KDa
35KDa

## Slide 6
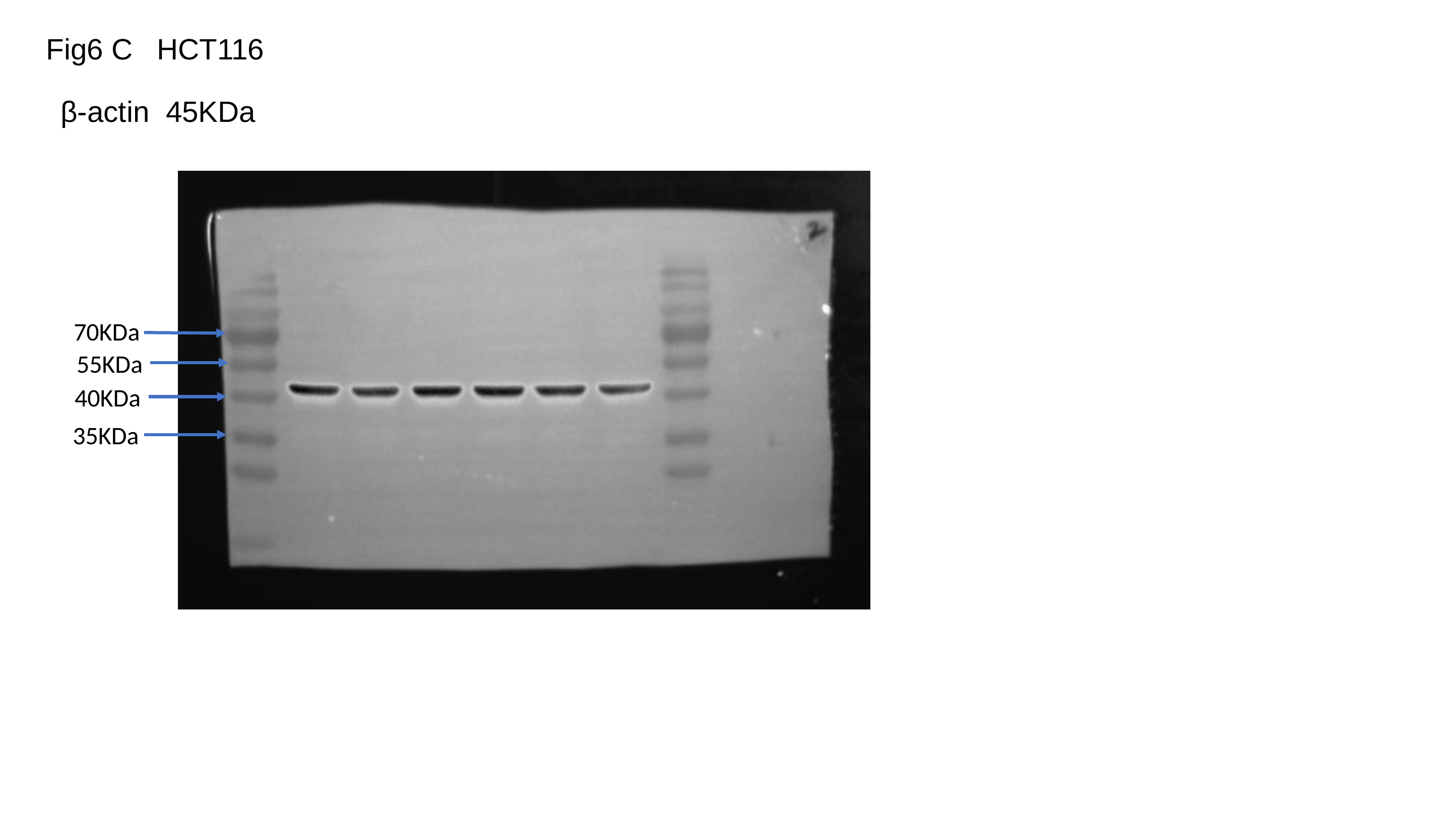

Fig6 C HCT116
β-actin 45KDa
70KDa
55KDa
40KDa
35KDa

## Slide 7
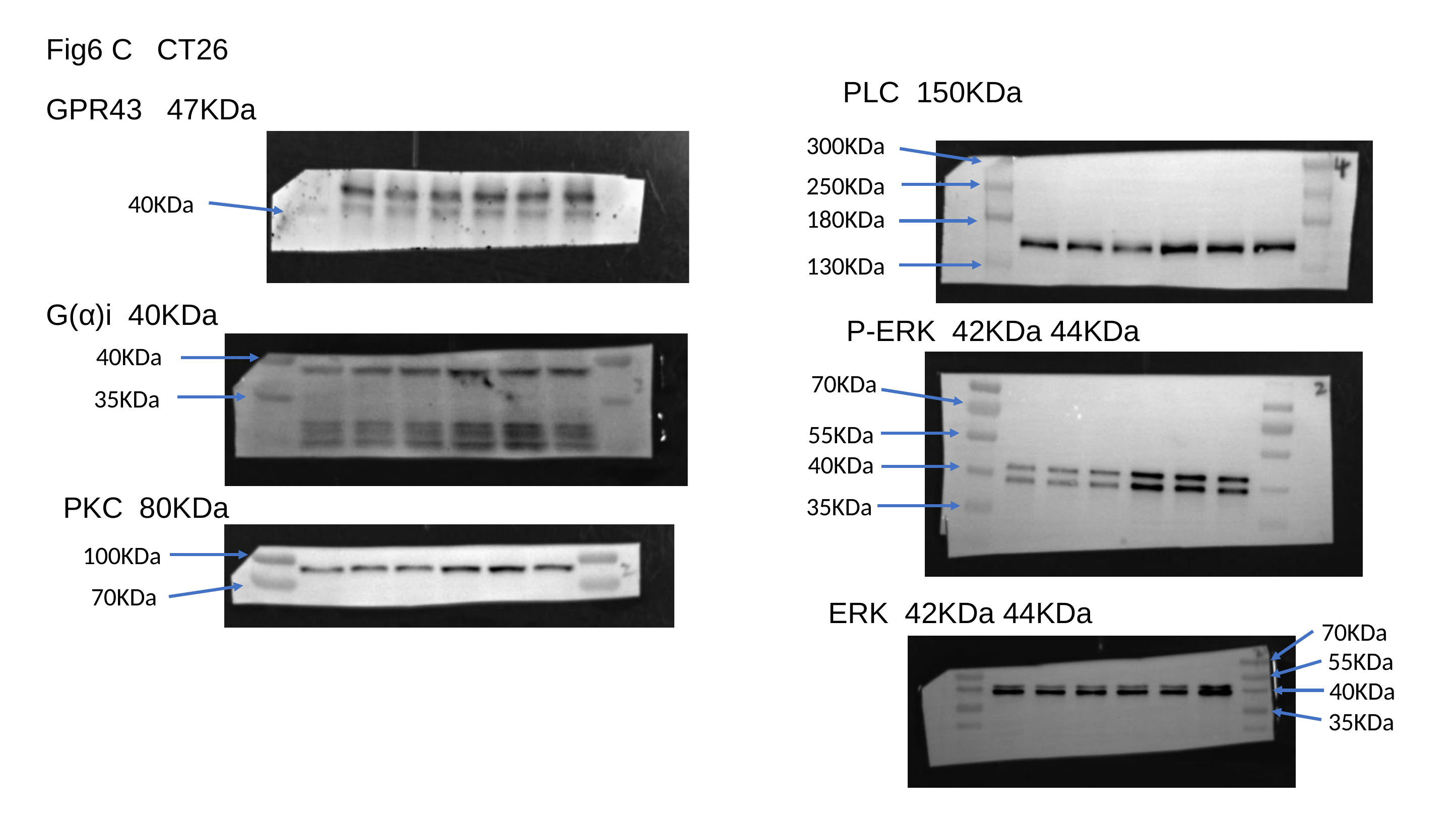

Fig6 C CT26
PLC 150KDa
GPR43 47KDa
300KDa
250KDa
40KDa
180KDa
130KDa
G(α)i 40KDa
P-ERK 42KDa 44KDa
40KDa
70KDa
35KDa
55KDa
40KDa
PKC 80KDa
35KDa
100KDa
70KDa
ERK 42KDa 44KDa
70KDa
55KDa
40KDa
35KDa

## Slide 8
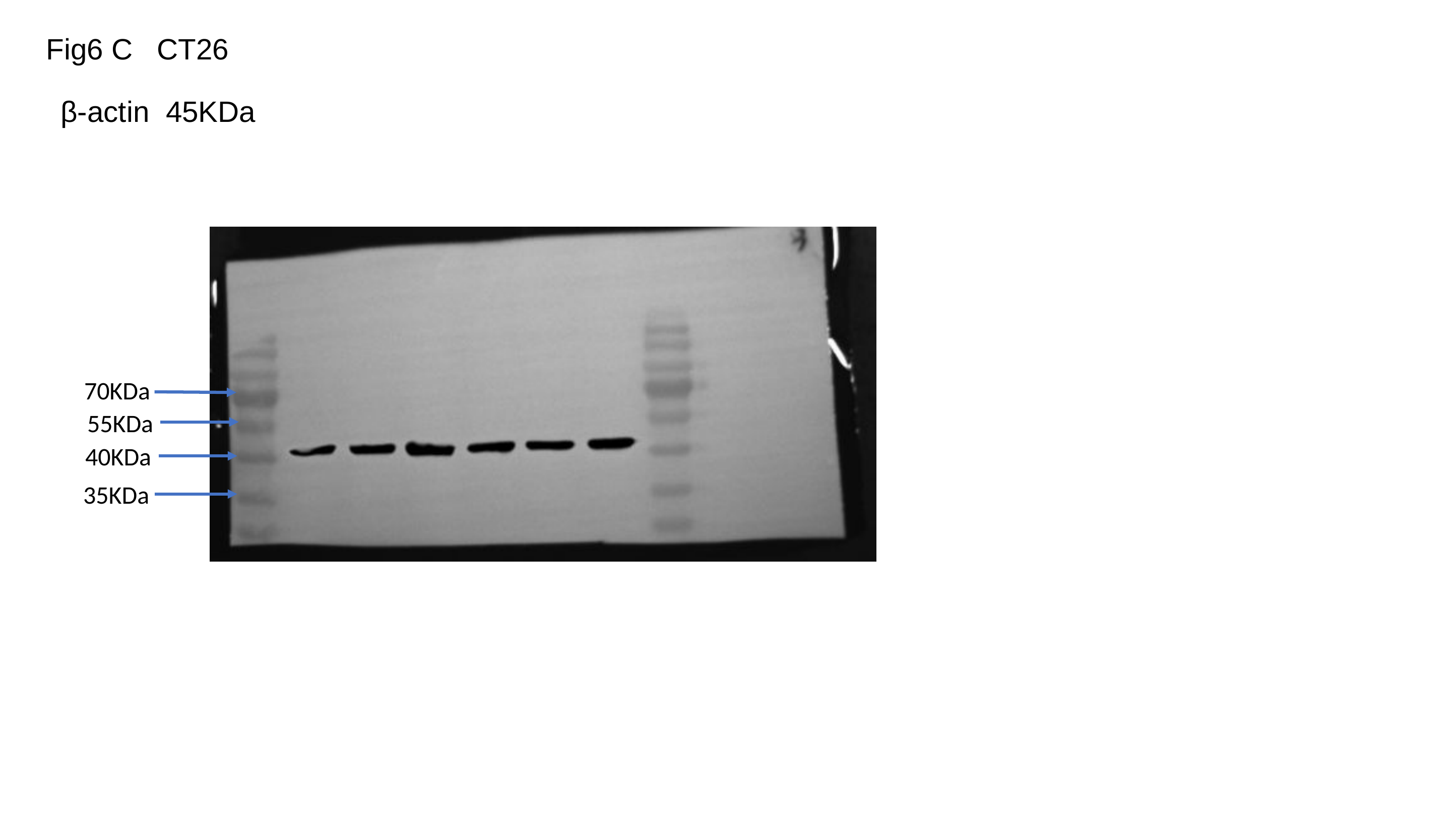

Fig6 C CT26
β-actin 45KDa
70KDa
55KDa
40KDa
35KDa
