# Supplementary material for: Bornlisy Attenuates Colitis-Associated Colorectal Cancer via Inhibiting GPR43-Mediated Glycolysis
Source: Front Nutr. 2021 Nov 12;8:706382. doi: 10.3389/fnut.2021.706382 (PMC8636091; doi:10.3389/fnut.2021.706382)
Supplement: Supplementary file 2 [file Data_Sheet_1.docx]

***Supplementary Information***


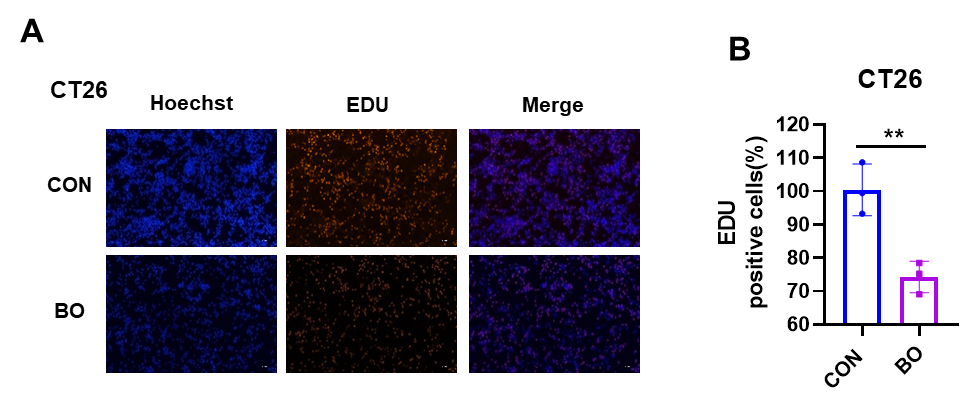


**Figure S1** **BO impairs proliferation and metastasis of CRC cells *in vitro*.** (A) CT26 cells were treated with BO (MOI=5, 24h). Cell proliferation was evaluated using immunofluorescence staining (scale bar, 100µm). (B) EdU-positive cells were calculated. Data with error bars are presented as mean ± SD. Each panel is a representative experiment of at least 3 independent biological replicates. * P <0.05, ** P < 0.01, ***p < 0.001 as determined by unpaired Student’s t test.


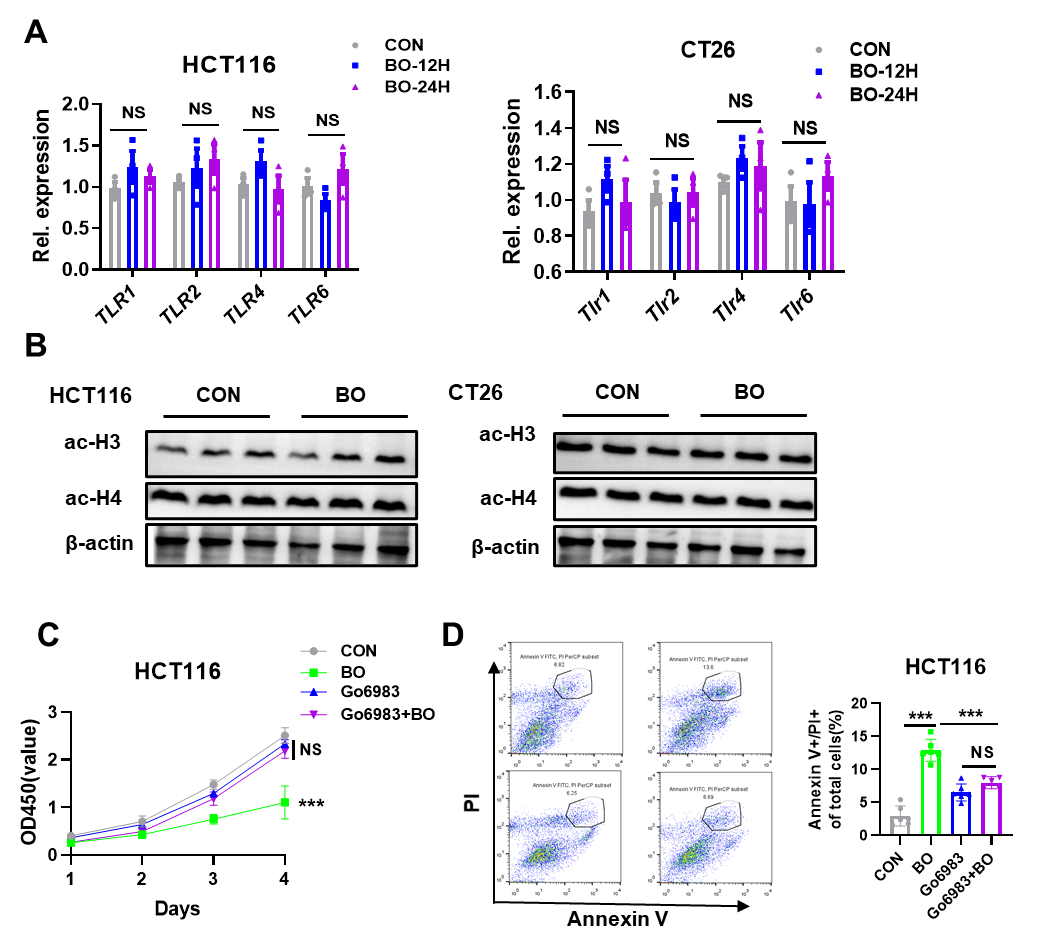


**Figure S2 GPR43 is a targeted receptor of BO in suppressing CRC proliferation.** (A) HCT116 cells and CT26 cells were treated with BO for 12h and 24h (MOI=5). mRNA expressions of *TLR1*, *TLR2*, *TLR4*, and *TLR6* in HCT116 cells and CT26 cells were detected by qPCR. (B) HCT116 cells and CT26 cells were treated with BO (MOI=5, 24h). Protein levels of ac-H3 and ac-H4 in HCT116 cells and CT26 cells were detected by western blots. (C-D) HCT116 cells were stimulated with BO (MOI=5, 24h) after pretreatment with Go6983 (10mM) for 4h. Cells proliferation was measured by CCK8 assay. Cell apoptosis was monitored by flow cytometry. The percentage of apoptotic cell (Annexin V^+^/PI^+^) were calculated. Data with error bars are presented as mean ± SD. Each panel is a representative experiment of at least 3 independent biological replicates. * P <0.05, ** P < 0.01, ***p < 0.001 as determined by unpaired Student’s t test.

**Supplemental Table 1. Histologic criteria of tumors. Related to Figure 1.**

| Histologic criteria of tumors |  | Score |
| --- | --- | --- |
|  | 0-2 | 1 |
|  | 3-5 | 2 |
|  | 6-8 | 3 |
|  | 9- | 7 |
| Crypts | normal | 0 |
|  | goblet cell depletion | 1 |
|  | branching | 2 |
|  | Complex budding | 3 |
| Epithelium | normal | 0 |
|  | Hyperplasia/aberrant crypt foci | 1 |
|  | low-grade dysplasia | 2 |
|  | High-grade dysplasia | 3 |
| Submucosal invasion | absent | 0 |
|  | present | 1 |

**Supplemental Table 2. Primers used for quantitative real-time PCR analysis**

| Gene | Forward Primers | Reverse Primers |
| --- | --- | --- |
| Mouse IL-6 | GAGGATACCACTCCCAACAGACC | AAGTGCATCATCGTTGTTCATACA |
| Mouse TNF-α | GCCTCCCTCTCATCAGTTCT | CACTTGGTGGTTTGCTACGA |
| Mouse c-Myc | CACCACCAGCAGCGACTCT | GGCACCTCTTGAGGACCAGT |
| Mouse GLUT1 | CAGTTCGGCTATAACACTGGTG | GCCCCCGACAGAGAAGATG |
| Mouse HK2 | CCCAGAGCCGGGTACAGAA | GGGGAGTTGGTCAGCTTCG |
| Mouse PKM2 | GCCGCCTGGACATTGACTC | CCATGAGAGAAATTCAGCCGAG |
| Mouse LDHA | CATTGTCAAGTACAGTCCACACT | TTCCAATTACTCGGTTTTTGGGA |
| Human c-Myc | GGCTCCTGGCAAAAGGTCA | CTGCGTAGTTGTGCTGATGT |
| Human GLUT1 | GGCCAAGAGTGTGCTAAAGAA | ACAGCGTTGATGCCAGACAG |
| Human HK2 | GAGCCACCACTCACCCTACT | CCAGGCATTCGGCAATGTG |
| Human PKM2 | ATGTCGAAGCCCCATAGTGAA | TGGGTGGTGAATCAATGTCCA |
| Human LDHA | ATGGCAACTCTAAAGGATCAGC | CCAACCCCAACAACTGTAATCT |
| Mouse TLR1 | TTTGTCCCACAATGAGCTAAAGG | TTCTTTGCATATAGGCAGGGC |
| Mouse TLR2 | CTCTTCAGCAAACGCTGTTCT | CTCTTCAGCAAACGCTGTTCT |
| Mouse TLR4 | ATGGCATGGCTTACACCACC | GAGGCCAATTTTGTCTCCACA |
| Mouse TLR6 | AGCCAAGACAGAAAACCCATC | GGGGTCATGCTTCCGACTAT |
| Human TLR1 | CCACGTTCCTAAAGACCTATCCC | CCAAGTGCTTGAGGTTCACAG |
| Human TLR2 | ATCCTCCAATCAGGCTTCTCT | GGACAGGTCAAGGCTTTTTACA |
| Human TLR4 | AGACCTGTCCCTGAACCCTAT | CGATGGACTTCTAAACCAGCCA |
| Human TLR6 | TTCTCCGACGGAAATGAATTTGC | CAGCGGTAGGTCTTTTGGAAC |
| Mouse GPR41 | GTGTAGTCTGTTGGTTCCTGG | TGTAGGTTGCATTTCCCCAG |
| Mouse GPR43 | gagcagctggatgtggtactg | catgggaacgaaaaacagga |
| Mouse GPR109a | TTTCTCCAGCCCATCTTTCC | ATACTTCTGGTTGTGCTGGG |
| Human GPR41 | TGCGGTCAATGGGATCTG | CAAGCACAGAGACGTAAGAGG |
| Human GPR43 | ttcaccgataaccagttggac | gggatgaagaagagcaccag |
| Human GPR109a | CACTCTCAGCTTCACCTACATG | CTCACCTGTCATCTTCCTCTG |
| Mouse β-actin | TACCACCATGTACCCAGGCA | CTCAGGAGGAGCAATGATCTTGAT |
| Human β-actin | CTGGCTCCTAGCACCATGAAGAT | GGTGGACAGTGAGGCCAGGAT |
